# What should neurologists expect to observe in relapsing polychondritis and VEXAS?

**DOI:** 10.3389/fimmu.2026.1811453

**Published:** 2026-04-24

**Authors:** Mickael Bonnan, Quang Tuan Rémy Nguyen, Etienne Crickx

**Affiliations:** 1 Neurology Department, Henri Mondor Hospital, Créteil, France; 2 Internal Medicine Department, Henri Mondor Hospital, Créteil, France; 3 Institut Mondor de Recherche Biomédicale (IMRB), INSERM U955, Université Paris Est- Créteil (UPEC), Créteil, France

**Keywords:** cranial nerve, encephalitis, meningitis, neurology, neuropathy

## Abstract

Relapsing polychondritis (RP) and VEXAS syndrome (Vacuoles, E1 enzyme, X-linked, Autoinflammatory, Somatic) are two clinically overlapping conditions in which neurological complications remain uncommon. Based on the existing literature, neurological involvement in RP most frequently comprises meningitis, encephalitis, cranial nerve palsies, and stroke, whereas VEXAS is additionally characterized by peripheral nerve and muscle involvement. This scoping review summarizes the neurological manifestations associated with RP and VEXAS and highlights the current gaps in knowledge regarding these complications.

## Highlights

- Neurological complications associated with RP and VEXAS are rare.
- The complication patterns are slightly different.
- Meningoencephalitis is often a presenting symptom of RP, with a severe course.
- With the exception of optic perineuritis, other cranial nerve palsies may be related to contiguous inflammation.
- Muscle and peripheral neuropathy could be more frequent in VEXAS.

## Introduction

Neurological manifestation of systemic disorders is more often discussed than observed in clinical practice, and typical clinical patterns constitute the main discussion guides. Relapsing polychondritis (RP) is a rare systemic autoimmune disease characterized by recurrent flare-ups of focal cartilage inflammation, such as ear, nasal or laryngotracheal/tracheobronchial chondritis, with no age or sex predominance (**Table 1**) (1–3). Other main symptoms include arthritis and ocular involvement, most commonly presenting as scleritis/episcleritis. In 2020, an acquired genetic mutation in *UBA1* was described as responsible for VEXAS syndrome (Vacuoles, E1 enzyme, X-linked, Autoinflammatory, Somatic), a discovery that quickly revealed substantial clinical overlap between both diseases (4). Main symptoms included recurrent fever, skin manifestations, chondritis, pulmonary involvement, and hematologic abnormalities, particularly macrocytic anemia. Notably, a subset of older, predominantly male patients previously classified as having RP associated with myelodysplastic syndromes and poor prognosis likely corresponded to patients with *UBA1* mutations (5, 6).

**Table 1:** Main features of RP and VEXAS.

Relapsing polychondritis	VEXAS
Any age or sex Main systemic signs: Bilateral auricular chondritis Nonerosive, seroneg. inflammatory polyarthritis Nasal chondritis Ocular inflammation Respiratory tract chondritis Cochlear or vestibular dysfunction or both Blood: SIS	Late onset, acquired X-linked mutation Main systemic signs: Fever Skin: neutrophilic dermatosis Rheumatology: arthritis Blood: macrocytic anemia, low platelets, SIS Lungs: alveolitis Ocular: orbital edema, scleritis Vascular: thrombosis Associated disorders: Sweet sd, polyarteritis nodosa, myelodysplastic sd, RP
Pathology: chondritis	Pathology: bone marrow vacuoles. UBA1 mutation

Sd, syndrome; SIS, systemic inflammatory syndrome.

Apart from systemic involvement (**Table 1**), RP is rarely accompanied by neurological lesions, which primarily manifest as cranial nerve palsies related to chondritis, and a combination of signs of meningitis and encephalitis. On the contrary, the neurological manifestations associated with VEXAS remain unclear and appear to be dominated by muscular and peripheral nerve involvement rather than central nervous system involvement. Vascular involvement may also occur in both diseases due to the interplay between vasculitis mechanisms and neurological involvement. Since neurological disorders are also more common in this aged population (e.g. Lewy body disease or Alzheimer/amyloid), neurologists may need to distinguish between multisystemic complications and fortuitous associations.

## Methods

A comprehensive search of several databases was conducted by the investigators in March 2025 without restriction on the time period or language (see detailed research strategy in **Supplementary Data 1**). Briefly, research strategy used combined keywords to search for neurological signs associated with ‘polychondritis’ or ‘VEXAS’. Diagnosis of RP or VEXAS were based on *ad-hoc* criteria (see **Supplementary Data 1**). One reviewer extracted the relevant data of the published cases. Case inclusion was based on the presence of neurological signs or symptoms (*e.g*. headache, cranial or peripheral nerve lesions, seizure, myositis, meningitis, encephalitis, or encephalopathy). Pure ocular myositis, or sensorineural hearing loss (SNHL), which are not solely neurological, were excluded due to their high frequency and the available reviews of these complications in RP and VEXAS (7, 8).

Relevant data of the study were: pattern of neurological signs and symptoms, cerebrospinal fluid (CSF) parameters, neuraxial lesions on magnetic resonance imaging (MRI), computed tomography (CT) scan or angiography, and pathology. No statistical analysis other than descriptive was performed. Data are given as rounded percentage (number) or median (interquartile range, IQR).

## Results

### Literature review

We collected 187 RP and 44 VEXAS cases with neurological signs or symptoms (**Figure s1** and raw data in **Supplementary Material**).

### Relapsing polychondritis

Yearly incidence of RP is estimated to 0.7 per million inhabitants and prevalence could be 4.5 per million (9, 10). RP is a rare autoimmune disease with male:female ratio 1:1, all ages being affected, median age 55 years. Although central nervous system (CNS) involvement is deemed to be rare (about 2% to 3% (11–13)), it may occur in 8% to 12% of large series (**Table 2**), reflecting more probably methodological differences. In a series of 239 RP, stroke occurred in 2%, leading to death in one case, meningitis/encephalitis in 5% (14), and 1% of incident RP were referred by neurologists. Neurological onset episodes or relapses are commonly associated with RP flare-ups (chondritis, fever or systemic symptoms) which may sometimes be missing. Headache may indicate aseptic meningitis, orbital or nasal inflammation. In the majority of cases, patients who had previously presented with unrecognized systemic symptoms subsequently developed neurological signs, leading to a retrospective diagnosis of RP at the time the neurological symptoms appeared. Others experienced a relapse of their chondritis within days of being hospitalized. However, complete absence of a history of RP at neurological onset was not uncommon, and these patients developed a full pattern within days after hospital admission.

**Table 2:** Frequency of neurological complications in RP and VEXAS.

Main clinical features	RP, % (n)	VEXAS, % (n)
Sex, M:F (n=148) Age, median [IQR] (n=148)	2:1 55 (19) [22 to 81]	Males 70 (14) [47 to 82]
Orbital lesions, % —Peri-orbital edema, %	4 to 6 ^a^ Absent	half ^e^ 8 to 30 ^f^
SNHL, %	9 to 28 ^b^	rare
Neurological signs, %	2 to 12 ^c^	2 to 15 ^g^
Neurological RP series	(n=187)	(n=68) ^h^
Neuropathy, % (n)	2 (3)	40 (27)
Myositis, % (n)	1 (2)	12 (8)
Cranial nerve, % (n) —VII, % (n) —V, % (n) —II, % (n) —oculomotor, % (n)	18 (33) 6 (11) 3 (5), mostly V1 4 (8), perineuritis 3 (6)	10 (7) 3 (2) 3 (2), perineuritis 4 (3), VI
Vascular, % (n)	10 (19)	18 (12)
—Vein thrombosis, % (n)	1 (1)	4 (3) ^i^
—Brain arteries, % (n)	7 (13)	12 (8)
—Brain aneurysm, % (n)	1 (2)	
—GCA (cervical), % (n)	0 (0)	2 (1)
Meningoencephalitis, % (n)	68 (128)	21 (14)
—Meningoencephalitis, % (n)	39 (72)	
—Encephalitis, % (n)	19 (35)	18 (12)
—Aseptic meningitis, % (n)	11 (21)	6 (4)
—Leptomeningitis, % (n)	10 (19)	
—Pachymeningitis, % (n)	6 (11)	3 (2)
—Enhancement, % (n)	25 (47)	
PRES, % (n)	1 (1)	4 (3)
Seizures, % (n)	10 (19)	4 (3)
Hallucinations, % (n)	13 (24)	
Others, % (n) ^d^	6 (12)	3 (2)^j^

Orbital lesions except cranial nerve paralysis were excluded. Patients may have multiple neurological signs. Most RP patients were diagnosed before the identification of VEXAS. GCA, giant cell arteritis; PRES, posterior reversible syndrome; SNHL, sensory neural hearing loss.

^a^ from (3, 8).

^b^ from (15).

^c^ from (1, 6, 11–14, 16, 60, 61).

^d^ Others: headache, seizure, brain pseudotumor, hydrocephalus, ataxia and SNHL, or unclear pattern.

^e^ from (50).

^f^ from (62, 63).

^g^ from (45, 47, 64).

^h^ orbital lesions except cranial nerve paralysis were excluded. Patients may have multiple neurological signs.

^i^ cerebral venous sinus (n=2), superficial temporal vein (n=1).

^j^ brain pseudotumor.

#### Cochleovestibular involvement

Cochlear and vestibular damage may give rise to vertigo, tinnitus and hearing loss which occurs in 9% at onset, and up to 28% of the followed patients in historical series (15). These signs are minor criteria of RP. Cochlear and vestibular attacks range from mild and transient to severe and definitive episodes. Sensorineural hearing loss (SNHL) is more frequent than conductive hearing loss. The mechanism could involve chondritis-induced lesion of the eustachian tube and inflammation of the inner ear. Although ataxia is frequently mentioned, it is more likely to be related to a vestibular cause, but there is no evidence for isolated damage for the VIII^th^ nerve or the cerebellum.

#### Ocular involvement

Ocular signs occur in 9% to 40% and up to 20% to 61% during follow-up patients in historical series (16). Common signs are anterior scleritis (or ‘redness’), conjunctivitis, proptosis, keratitis, uveitis or retinopathy. Optic neuropathy might occur in 4% to 6% and may relapse. Ischemic neuropathy is often advocated in relation with systemic vasculitis, however supposed examples of ischemic mechanisms are rare and poorly supported by available data. The most common mechanism is an optic perineuritis with papilledema, occurring in association with scleritis or orbital myositis (17). Only one case displayed unilateral focal optic tract inflammation (18). Finally, since the use of MRI, no cases of isolated retrobulbar optic neuritis or optic ischemia have been diagnosed. One case of recurrent unilateral ‘optic neuritis’ revealed a compressive mechanism related to a periostitis of the optic canal, although p-ANCA(+) basal pachymeningitis remained possible (19). Extraocular muscle nerve palsy occurs in 4%, with medial or lateral rectus muscles being often involved (20), although the commonly associated orbital signs (e.g. proptosis, exophthalmia) pointed to myositis or orbital cellulitis, which are often confirmed by MRI in recent cases, rather than true nerve lesion.

#### Cranial nerves involvement

Cranial nerve palsy is often mentioned in textbooks and facial weakness has been reported in 2% of RP, and is dominated by extrinsic involvement of the cranial nerves. Few cases have developed bilateral paralysis of nerve VII and VIII (pseudo-Ramsay-Hunt syndrome) in association with severe chondritis. Other cases reporting signs of cranial paralysis or facial sensory disturbances are rare and without reliable mechanism (compression by facial edema or chondritis being probable), or remain intertwined with central lesions. Lesion of mixed cranial nerves (IX, X) and XII nerve are rare and intertwined with pharyngeal chondritis. Peripheral neuropathy is highly uncommon (2%): two polyneuritis and one mononeuritis multiplex were reported. A historical series of 119 cases mentions without detail a case of sensorimotor polyneuropathy and a mononeuritis multiplex (8).

#### Vascular involvement

Vascular complications occur in less than 15% of RP, including aortic arch aneurysms, whereas involvement of cerebral arteries remains rare. Vasculitis, which frequently affects the aorta, can also affect all large and medium-sized vessels, including cerebral arteries, and aortic arch lesions were the leading cause of death during follow-up. Among other vessels, thrombosis of cerebral arteries or veins accounts of 10% (n=19) of neurological cases. Strokes can be caused by vasculitis, but other common mechanisms should not be overlooked in elderly patients or those with poorly documented medical histories. Imaging of brain arteries by CT angiography or by arteriography acquired in meningoencephalitis patients has not revealed arteritis. Only two cases were related with proven vasculitis: one with mild bilateral carotid stenosis remitting after corticosteroids, and one with an enhanced arterial wall in intracranial large vessels (21, 22). Aortic arch is involved in 11% (23), while a painful extracranial fusiform carotid aneurysm has been reported (24). Conversely, cranial vessels remain mostly spared by aneurysms, since fortuitous, uncomplicated aneurysms were only twice reported in intracranial arteries (25, 26).

#### Meningoencephalic inflammation

Confusion and encephalopathy are associated with meningoencephalitic episodes, which may be single or recurrent multiple times (up to 6 times). The episodes are accompanied by recurrent CSF pleocytosis. Focal seizures and hallucinations were observed in 10% (n=19) and 13% (n=24) respectively. Meningoencephalitis was observed in 68% at age 57 (IQR 22; 22 to 81 years), with M:F ratio 3:1. Although imprecise retrospective data impede fine clinical diagnosis, proportion of meningoencephalitis, encephalitis and aseptic meningitis was roughly 4:2:1. Aseptic meningitis is mostly associated with isolated headaches responding within days to corticosteroids. Although CSF pleocytosis is common, biopsy-proven encephalitis were observed in RP patients with normal CSF (27, 28).

A subset of papers providing detailed data allowed to distinguish different patterns of encephalitis: a) limbic encephalitis was defined by subacute onset (<3 months) of memory deficits, or psychiatric symptoms, and bilateral MRI lesions predominating in medial temporal lobes (29); b) striatal encephalitis: onset with sleepiness, cognitive disturbance, movement disorders or parkinsonism (27, 30, 31); c) mixed limbic and striatal encephalitis; d) cortical encephalitis (32); or e) non-specific white matter (WM) encephalitis including corpus callosum.

Clinical signs of limbic or striatal encephalitis could be reverted by high dose corticosteroids with late atrophic change of the caudate, or worsened despite treatment. However, cognitive decline is not always stopped by treatment, resulting in severe temporal atrophy, probably related to corticosteroid-resistant encephalitis.

Lepto- or pachymeningitis was observed in 14 cases with various patterns of lesions: diffuse or combining falx, frontal-temporal, tentorium cerebelli or spinal locations. In six cases, PR3-ANCA, IgG4 or plasma cell granuloma were demonstrated, and one case was a pseudotumoral *en plaque* lesion. Two cases also showed restricted CSF diffusivity (33), as has been reported in rheumatoid meningitis. The CSF showed variable numbers of lymphocytic white blood cells (WBC), and intrathecal IgG synthesis was observed in half of the four CSF examined. In contrast, RP is absent from large series of hypertrophic pachymeningitis (HP) (34), confirming that RP remains a rare cause of HP, often associated with comorbid disorders. Spinal cord involvement remains uncertain.

#### Cerebrospinal fluid and blood

CSF WBC count was reported in 67% of cases, of which 82% had meningitis (>5 WBC per mm^3^) (**Table 3**). Lymphocyte predominance was observed in 75%, whereas polymorphonuclear predominance was associated with higher WBC count (median 52 vs 468 WBC). Proteins were elevated in 72% of cases, and 18% had values above 1g/L. CSF glucose remained near normal, including with elevated CSF WBC count. Rare cases presented with low CSF glucose (1.5 to 2.1 mmol/L) despite low cell counts, and two recurrent cases presented with very low glucose (1 to 2.1mmol/L) with prominent WBC count (1,700 to 4,000 WBC, predominantly polymorphonuclear, PMN) in the absence of infection. A normal CSF count on lumbar puncture (LP) does not exclude meningeal inflammation confirmed by biopsy or autopsy (35), and CSF WBC counts also returned to normal within a few days after Cs. One case with severe basal ganglia meningoencephalitis converted from early lymphocytic meningitis to eosinophilic meningitis (219 WBC, 16% eosinophils) (36). Intrathecal synthesis was positive in 76% of the CSF examined (n=21), either the IgG index or oligoclonal bands (OCB), and all but two had elevated WBC counts. CSF IL6 levels are mildly or markedly elevated even without meningitis, while CSF TNFα remains negative. Based on these cases, the consistent elevation of CSF IL6 supports a role for drugs targeting IL6, whose CSF levels are effectively normalized by the treatment of encephalitis. Anti-neuronal antibodies were often negative, except for anecdotal reports of anti-NR2B. CRP and erythrocyte sedimentation rate (ESR) may be normal or moderately elevated.

**Table 3:** Cerebrospinal fluid features in RP.

CSF	n=126
—Meningitis (>5 WBC), % (n)	82 (104)
—Lymphocyte predominant, % (n) WBC, median (IQR) [min-max] Lymphocytes, % (IQR)	75 (62) 52 (124) [6 to 800] 79 (34)
—PMN predominant, % (n)	25 (21)
WBC, median (IQR) [min-max] Polymorphonuclear, % (IQR)	468 (761) [45 to 4000] 81 (20)
—High CSF proteins (≥0.5g/L), % (n) ≥1 g/L, % (n)	72 (78/108) 18 (19) [up to 5.8 g/L]

Patients had two (n=8) or three (n=1) LP. WBC values were obtained for 83 LP. LP were done in patients with meningitis/encephalitis signs in 90%, and 94% (n=98) of the biological meningitis were congruent with clinical signs. Ly, lymphocytes; PMN, polymorphonuclear; WBC, white blood cell count.

#### Imaging

Whole body FDG-PET usually shows uptake in chondral areas (nose, pharynx, bronchi) suggesting chondritis, while decreased uptake in the cortex is associated with cognitive impairment. Increased uptake has been observed in the hippocampus during encephalitis (37). Brain MRI shows restricted diffusion and enhancement of auricular chondra (*prominent ear sign*), which disappears a few days after corticosteroids (38).

Brain lesions mainly involved supratentorial areas, with the exception of a few cerebellar lesions. Non-specific localized, multifocal hyperintense patchy lesions are frequently observed on T2-wheighted sequences. Enhancement occurred in 25% (n=47) of cases, combining involved areas of the WM, cortex or basal ganglia, or were localized in the leptomeninges. Partial regression of WM lesions could be achieved within days following corticosteroids. Brain atrophy, particularly of the medial temporal cortex, was reported or obvious in 90% (n=27) of encephalitis, and could develop within the first months after the onset of encephalitis, either symmetrically or asymmetrically. On the contrary, radiological signs of encephalitis could remain below the level of clinical expression and disappear after corticosteroids administration.

#### Pathology of CNS lesions and pathophysiology

Pathology was based on autopsy (n=8) or brain/meningeal biopsy (n=15). Meningeal inflammation was always observed, mainly T cells, abundant CD20+ B cells, PMNs, eosinophils, and granulomas. Perivascular cuffing was frequently observed in brain biopsies, whereas vasculitis and thrombosis were rarely detected by biopsy (39, 40). Mild to extensive necrosis of the brain parenchyma may be observed. Autopsy confirms leptomeningitis, perivascular cuffing in the brain parenchyma, perivascular demyelination and microglial nodules. Necrotizing arteritis and vasculitis with venous or arterial thrombosis are rare. Muscle biopsy during meningoencephalitis and myositis once showed a vasculitic process (36).

The authors hypothesized that meningeal antigens such as collagen might trigger the meningoencephalitis because antibodies against type II collagen, and possibly against types IX and XI, are the hallmark of RP. In the CNS, type II collagen is expressed in hippocampus, thalamus and Schwann cells, whereas types IX and XI are expressed in meninges and frontal lobes (41). Although never studied, the demonstration of intrathecal synthesis of antibodies against collagen would provide very valuable clues.

Anti-neutral glycosphingolipid antibodies (GlcCer and GalCer) were transiently recovered from the sera of RP patients with limbic encephalitis, but not in controls, and then disappeared after clinical improvement. Neutral glycosphingolipid are expressed at high level in the pig ear cartilage. Unfortunately, apart from these few patients (42), this seminal work has not been replicated, and these antibodies were eventually also observed in non-RP limbic encephalitis (43). Finally, the degree of similarity between chondral collagen epitopes and meningeal collagen fibers is unclear.

### VEXAS

While RP is considered a rare disease, VEXAS was unexpectedly and widely found after systematic genetic testing identified mutations in 1 in 4269 men over the age of 50 (44). Among patients suffering from RP, male sex, and either mean corpuscular volume >100 fL or platelet count <200 G/L are highly predictive of VEXAS (5). In a series of 89 patients, cranial symptoms were observed in 15.7%, with 13% headache, and jaw pain, vision change or abnormal temporal artery in 2% each (45). Among 14 Japanese VEXAS patients, meningitis and SNHL occurred in one, stroke during cardiac resuscitation in one, and one had headache leading to the discovery of giant cell artery (GCA) (46). In a series of 44 patients enrolled in hematopoietic stem cell transplantation (HSCT) trial, one (2%) presented neurological signs at onset, and 4 (9.1%) during follow-up (47). Finally, neurological lesions are uncommon in VEXAS (**Table 2**). Cause of death in VEXAS is unrelated to neurological involvement, but occurs in 13% (n=6) of the series.

#### Ocular involvement

Ocular signs were observed in half of the patients included in the seminal description (4) and in 13-15% of larger series (48, 49). Orbital lesions are the presenting symptom in a quarter. Bilateral eye involvement is observed in half of the cases, although this does not usually occur at the same time. Periorbital edema is the most common sign, occurring in 8.6 to 30%, and becomes almost constant in patients reported by ophthalmologists. Observed signs could be ptosis, orbital inflammation, fat infiltration or pseudotumor, orbital cellulitis, myositis, or dacryoadenitis. Rarely, orbital edema leads to orbital compartment and decrease of visual acuity. Ocular globe is commonly involved by conjunctival injection, anterior or posterior scleritis, or anterior uveitis. Orbital imaging may demonstrate intraconal muscle and lacrimal gland enlargement, and preseptal swelling (or cellulitis) (50). Orbital biopsy is often non conclusive and show non-specific inflammation. Diplopia is secondary to orbital edema or focal myositis of extraocular muscles, especially involving the medial rectus muscle. Superior orbital fissure syndrome (Rochon-Duvigneaud) may occur in combination with myositis and nerve inflammation, or posterior extension of inflammation to cavernous sinus. However, it remains difficult to distinguish between myositis (or focal inflammation) and nerve paralysis, especially since sensory nerve branches seem to be spared. Although optic neuritis, retinal vasculitis and ischemic optic neuropathy (in association with large or small vessel vasculitis) were once described, ocular lesions observed in VEXAS are not expected to decrease visual acuity in isolation. Nerve inflammation, characterized by enlargement and signal change, is rarely confirmed by MRI (51). Other cranial nerves are spared, and only one trigeminal neuralgia was reported.

#### Peripheral nervous system involvement

Although neuropathies account of 40% (n=27) of the neurological feature associated with VEXAS, no specific pattern emerges from the cases: acute-onset CIDP, distal or non-length dependent, sensory or motor, axonal or demyelinating polyneuropathies could be observed. Vasculitis was proposed in a case associated with polyarteritis nodosa. Among the five nerve biopsies, none of them showed any VEXAS-associated inflammation. It remains unclear whether neuropathies are actually related to VEXAS until a larger series of pathological examinations has been conducted. Extraocular muscles were involved in 18.2% (n=8), mostly in lower limbs with fasciitis, or multifocal myositis with normal creatine kinase (CK) (52). Trismus may reveal myositis in the masticator space in the absence of GCA.

#### Vascular involvement

Thrombosis is increasingly recognized in VEXAS, with incidence of venous thrombosis being 35% to 56% and incidence of arterial thrombosis 1% to 25% (review in (53)). Among the series, 18% (n=12) developed cranio-cervical thrombosis: venous (n=3) either brain sinus or temporal veins; arterial (n=8) with stroke; or GCA (n=1) involving cervical arteries. Thrombosis usually occurs spontaneously and can also occur during anticoagulation. Mechanisms remain unclear and could combine inflammatory state, exaggerated NETosis, vasculitis and stasis. Periarterial thickening or parietal inflammation of carotid arteries was observed in two VEXAS patients and neutrophilic inflammation of temporal artery in one. It is interesting to note that all three patients with large vessel vasculitis displayed a thickening of the common carotid artery wall, which remains highly uncommon in GCA. Cerebral (n=2) or cranial (n=1) veins remain uncommon sites of thrombosis. Vein thrombosis may indicate a Behçet-like manifestation of VEXAS, which may occur in patients already receiving immunosuppressive drugs, and unrelated to the possible thrombotic side-effect of JAK inhibitors (54).

#### Encephalic and meningeal involvement

Patterns of encephalitis still remain unclear in VEXAS. Seizures were reported. Meningitis or encephalitis could occur in 21% (n=14): encephalitis or encephalopathy (n=11), post-infectious acute disseminated encephalomyelitis (ADEM)-like (n=1), meningoencephalitis (n=1), aseptic meningitis (n=2) and pachymeningitis (n=2). WBC count was normal in four and CSF IL6 was elevated in one. All encephalitic patients had white matter lesions, but lack of data and follow-up makes it impossible to distinguish related lesions. A brain MRI follow-up was reported in one, showing complete normalization.

## Discussion

**Figure 1:**
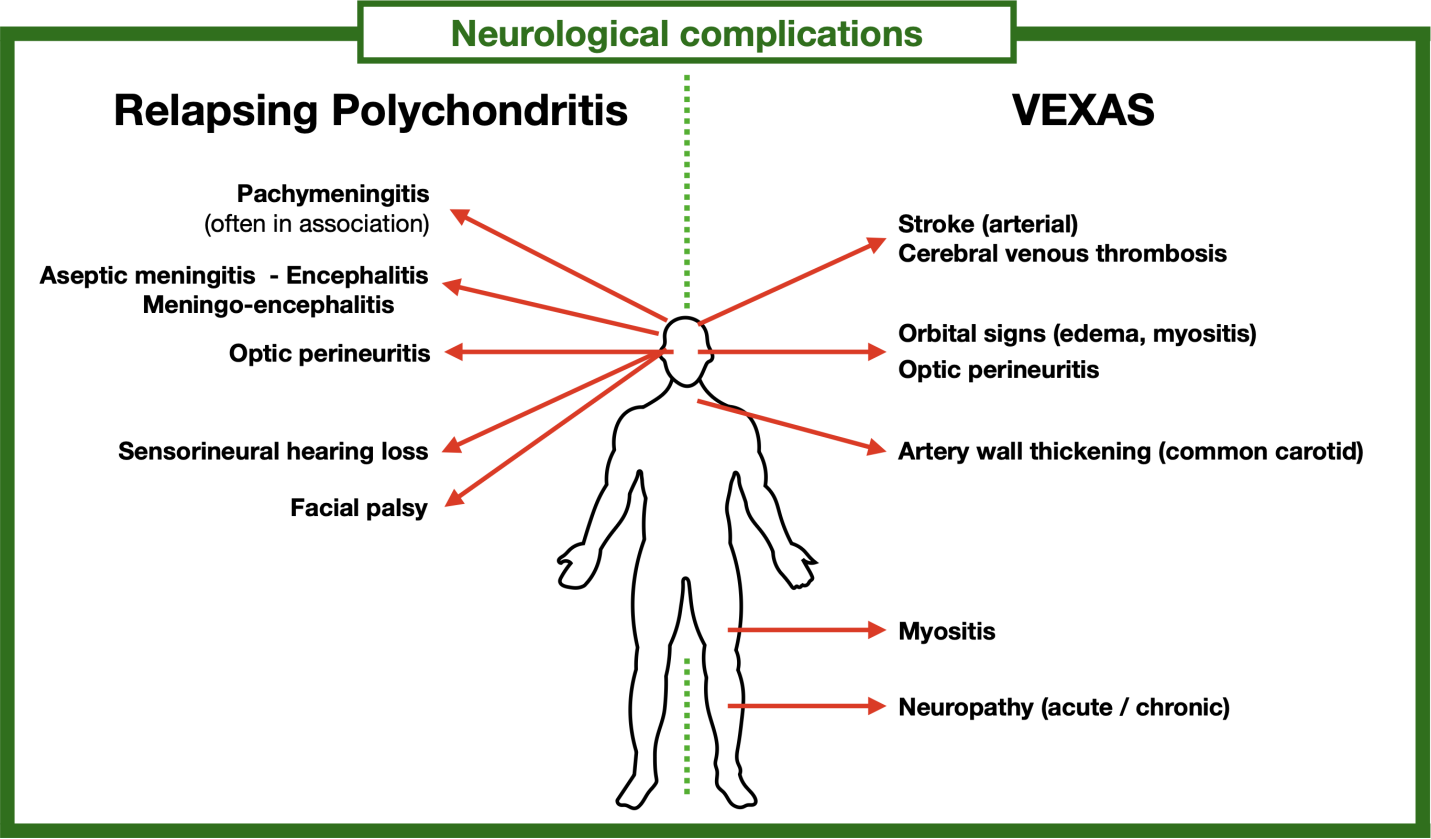
Main neurological complications of RP and VEXAS.

The main interest of this work is to identify the clinical patterns of neurological involvement associated with RP and VEXAS, allowing easier recognition (**Figure 1**).

In a large series of pre-VEXAS RP, multiple component analysis delineated several clusters characterized by distinct neurological profiles (6). A cluster of patients likely to have undiagnosed VEXAS cases (male, severe outcome, multisystem involvement, myelodysplasia) predominantly had PNS lesions, while another cluster of RP patients (68% women, younger, low severity, good response), predominantly had CNS involvement (6), supporting the hypothesis of different patterns of neurological complications between RP and VEXAS.

Data regarding pathophysiology of neurological involvement in both diseases are limited. In RP, autoimmune response with evidence of both humoral and cellular immune mechanisms have been proposed to mediate CNS inflammation, with rare reports of anti-gamma-aminobutyric acid B receptor autoantibodies and elevated cerebrospinal fluid cytokines (55, 56).

Interestingly, mutations in genes coding for enzymes involved in the ubiquitination cascade are associated with many neurodegenerative diseases (57), but *UBA1* mutations in VEXAS are somatic and restricted to myeloid cells while resident brain cells remain spared (58). However, it is possible that mutated inflammatory cells migrate to CNS/PNS and directly contribute to neurological lesions, such as described in the skin (59). So far, there is a lack of CSF data in VEXAS.

This review has some limitations. We cannot exclude that same patients could have been described in different articles, especially if they have been reclassified to VEXAS in later studies, which could have attenuated differences between VEXAS and non-VEXAS RP patterns. Direct comparison of lesion patterns is limited due to the unavailability of the original images and the brevity of captions. The lack of major clinical data prevented precise clinical distinction (*e.g.* encephalitis and encephalopathy). However, this review is the first to attempt to clarify the neurological complications of RP and VEXAS. This retrospective work is not intended to provide incidence data, given that it is based on retrospective studies. Moreover, published cases are biased toward rare and atypical features that are likely overrepresented in the literature, while signs less clearly related to VEXAS could be dismissed as unrelated complications (*e.g*. non-specific white matter lesions). Importantly, RP can overlap with other autoimmune diseases with neurological involvement, most commonly systemic vasculitis, rheumatoid arthritis, systemic lupus erythematosus, or autoimmune thyroiditis. Consequently, it is challenging to determine whether all neurological complications described in this review are specifically attributable to RP.

In conclusion, while RP and VEXAS share overlapping clinical features, neurological involvement follows distinct patterns in each disease. Encephalitis associated with RP is a serious condition that may precede systemic manifestations, whereas it appears to be uncommon in VEXAS. In contrast, neuromuscular involvement is considerably more frequent in VEXAS. International multicenter studies specifically dedicated to this topic are needed to validate and refine these observations.
