# Supplementary material for: What should neurologists expect to observe in relapsing polychondritis and VEXAS?
Source: Front Immunol. 2026 Apr 24;17:1811453. doi: 10.3389/fimmu.2026.1811453 (PMC13152779; doi:10.3389/fimmu.2026.1811453)
Supplement: Supplementary file 1 [file DataSheet1.docx]

Supplementary Material 1

**What should neurologists expect to observe in relapsing polychondritis and VEXAS?**

**BONNAN, Mickael ^1^, NGUYEN, Quang Tuan Rémy ^1^, CRICKX, Etienne ^2,3^**

^1^ Neurology department, Henri Mondor Hospital, AP-HP, Créteil, France; ^2^ Internal Medicine Department, Henri Mondor Hospital, AP-HP, Créteil, France; ^3^ Institut Mondor de recherche biomédicale (IMRB), INSERM U955, Université Paris Est- Créteil (UPEC), Créteil, France

***** Correspondence: mickael.bonnan@aphp.fr, Tel.: +33(0)1 49 81 23 04

# Supplementary Data. PRISMA Flow Diagram.

A comprehensive search of several databases (Scopus, Ovid, Willey, Karger, Pubmed and a database of French PhD thesis) was conducted by the investigator in March 2025 without restriction on the time period or language. MEDLINE database was searched up to May 2025. The search strategy was designed to broadly include neurological signs associated with polychondritis or VEXAS. PubMed search: (polychondritis OR VEXAS) AND (mening* OR brain OR cranial OR spinal OR muscle OR myositis OR stroke OR epilepsy OR dementia OR confusion OR encephal* OR neuro* OR nerve). List of associated results was manually reviewed for additional cases.

All abstracts of selected articles were read. All articles dealing with the questions raised in the introduction were carefully read and eventually used in the literature review. References of articles were also examined for unnoticed related articles. Authors were not contacted for additional references. Cases with doubtful RP/VEXAS diagnosis due to unavailable key clinical or biological data, and duplicate cases were discarded.


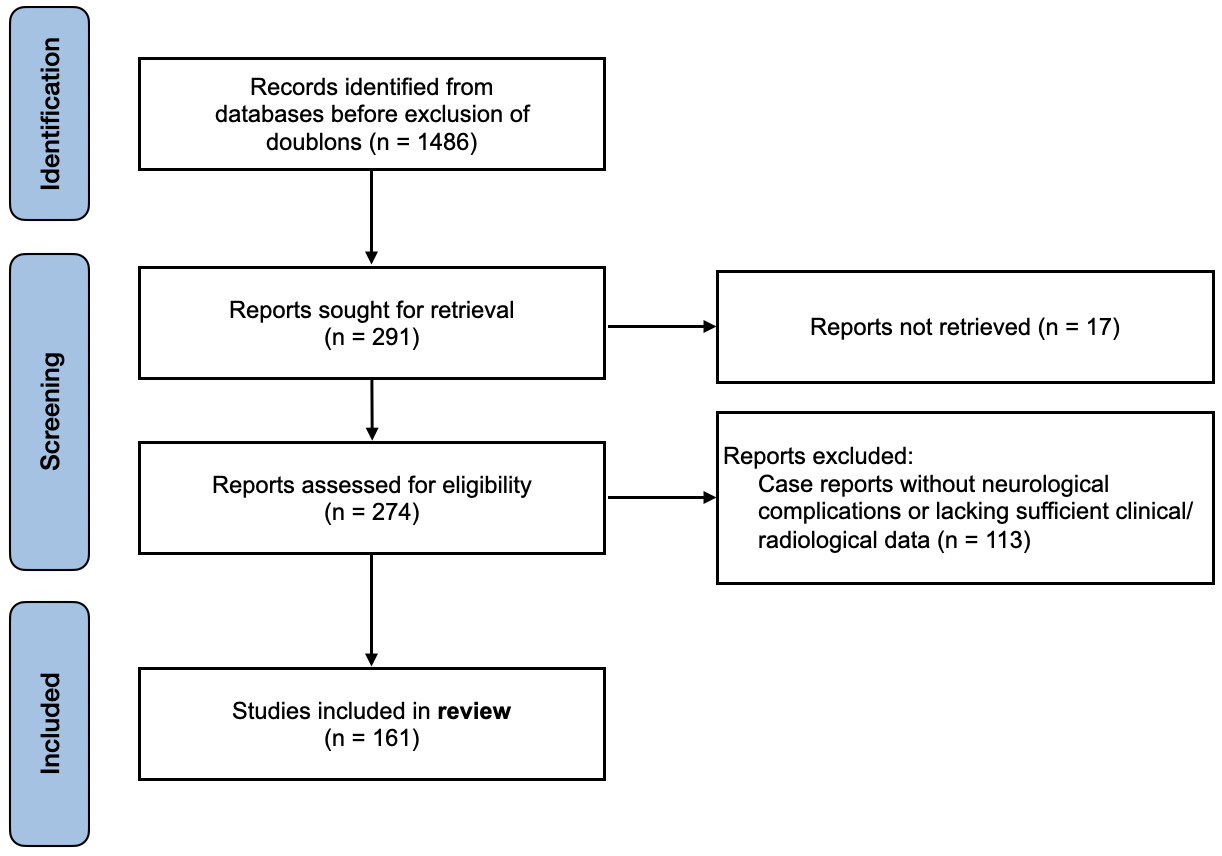


**PRISMA Flow diagram**

# Diagnosis criteria for relapsing polychondritis (RP).

**Criteria for diagnosing relapsing polychondritis** (critères de Michet et al., 1986).

**Major criteria**

Inflammation in the auricular cartilage

Inflammation in the nasal cartilage

Inflammation in the laryngotracheal cartilage

**Minor criteria**

Conjonctivitis, episcleritis, scleritis, or uveitis

Hearing loss

Vestibular dysfunction

Seronegative polyarthritis

*Two major criteria or one major criteria + two minor criteria are needed to classify the patient as having relapsing polychondritis.*

**Clinical features (McAdam, Damiani [33,194])**

Bilateral auricular chondritis

Nonerosive, seronegative inflammatory polyarthritis

Nasal chondritis

Ocular inflammation

Respiratory tract chondritis

Cochlear or vestibular dysfunction or both

*—Diagnosis (one of the following):*

*Three clinical features, or*

*One clinical feature and histologic evidence of chondritis*

*Chondritis at two or more separate anatomical locations, with response to corticosteroids, dapsone, or both*

# Figure s1. Mean yearly published neurological complications of relapsing polychondritis (RP) and VEXAS. RP and VEXAS diseases were identified respectively in 1923 and 2020. Neurological complications of VEXAS are now more frequently reported than those of RP.
