# Supplementary material for: What should neurologists expect to observe in relapsing polychondritis and VEXAS?
Source: Front Immunol. 2026 Apr 24;17:1811453. doi: 10.3389/fimmu.2026.1811453 (PMC13152779; doi:10.3389/fimmu.2026.1811453)
Supplement: Supplementary file 2 [file DataSheet2.docx]

Supplementary Material 2

**What should neurologists expect to observe in relapsing polychondritis and VEXAS?**

**BONNAN, Mickael ^1^, NGUYEN, Quang Tuan Rémy ^1^, CRICKX, Etienne ^2,3^**

^1^ Neurology department, Henri Mondor Hospital, AP-HP, Créteil, France; ^2^ Internal Medicine Department, Henri Mondor Hospital, AP-HP, Créteil, France; ^3^ Institut Mondor de recherche biomédicale (IMRB), INSERM U955, Université Paris Est- Créteil (UPEC), Créteil, France

***** Correspondence: mickael.bonnan@aphp.fr, Tel.: +33(0)1 49 81 23 04

**Supplemental Table. Literature review: data summary and references.**

**Abbreviations:** ADEM: acute disseminated encephalomyelitis, Aza: azathioprine, CN: cranial nerve, Cs: corticosteroids, CSF: cerebrospinal fluid, CT: computerized tomography scan, Cyc: cyclophosphamide, E: encephalitis, GBS: Guillain-Barré syndrome, GCA: giant cell arteritis, GM: gray matter, HCQ: hydroxychloroquine, HS: high signal, IS: immunosuppressive drugs, L: left, Leflu: leflunomide, leptoM: leptomeningitis, LL: lower limbs, LM: leptomeningeal, Ly: lymphocytes, M: meningitis, ME: meningoencephalitis, MMF: mofetil mycofenolate, MTX: methotrexate, nd: no data, OCB: oligoclonal bands, pachyM: pachymeningitis, PLEX: plasma exchange, PM: pachymeningeal, PMN: polymorphonuclear, PNP: peripheral neuropathy, PRES: Posterior reversible encephalopathy syndrome, R: right, SNHL: sensorineurl hearing loss, TCZ: tocilizumab, UL: upper limbs, WBC: white blood cell count, WM: white matter.

| **Authors** | **Year** | **Diag** | **Age** | **Sex** | **Signs** | **Syndrome** | **MRI T2/FLAIR** | **MRI contrast** | **Atrophy** | **CSF WBC (% Ly)** | **CSF prot (g/L)** | **Intrathecal IgG synthesis** | **Drugs** | **Response** | **Relapse (neurological)** | **Note** |
| --- | --- | --- | --- | --- | --- | --- | --- | --- | --- | --- | --- | --- | --- | --- | --- | --- |
| Rucker[1] | 1965 | RP | 41 | F | ocular pain, proptosis, photophobia, mild unilat blurring, VI | CN |  |  |  |  |  |  |  |  |  |  |
| Herman[2] | 1973 | RP | 55 | M | ischemic optic neuritis, middle cerebral artery thrombosis | stroke, CN |  |  |  |  |  |  | Cs, Aza |  |  |  |
| Herman[2] | 1973 | RP | 57 | M | polyneuritis, vertigo | PNP |  |  |  |  |  |  |  |  |  |  |
| McAdam[3] | 1976 | RP | 33 | M | L VII then bilateral, papilledema | CN |  |  |  | normal |  |  |  |  |  | autopsy: necrotizing arteritis |
| McAdam[3] | 1976 | RP |  |  | Bilateral facial palsy | CN |  |  |  |  |  |  |  |  |  |  |
| Ridgway[4] | 1979 | RP | 36 | F | headache, diplopia, vertigo | ME | nd | craniotomy: ependymoma |  | normal |  |  | Cs | partial | death | septicemia and brain tumor |
| Ridgway[4] | 1979 | RP | 52 | M | carotid stenosis, retinal embole | vasculitis ? Atheroma? |  |  |  | nd |  |  | Cs |  |  | endarteriectomia |
| Ridgway[4] | 1979 | RP | 81 | F | headache, SNHL | unclear |  |  |  | normal |  |  | dapsone | yes | no |  |
| Sundaram[5] | 1983 | RP | 58 | F | confusion, cochlear and vestibular signs | unclear, SNHL | CT: midbrain hypodensity |  |  | normal |  |  | Cs | yes | yes (general) |  |
| Hull[6] | 1984 | RP | 51 | M | ataxia, cochlear and vestibular signs, facial weakness, myopathy | "vasculitis" ? | CT: cerebellar stroke |  | yes | normal |  |  | Cs | no | deterioration |  |
| Willis[7] | 1984 | RP | 64 | F | Bilateral facial palsy | CN |  |  |  |  |  |  | Cs | yes | no |  |
| Serratrice[8] | 1986 | RP | 49 | F | fronto-orbital paresthesia (right V1) | CN |  |  |  |  |  |  | Cs | yes |  |  |
| Brod[9] | 1988 | RP | 30 | F | ataxia, headache | ME |  |  |  | 19-37 (Ly) |  |  | nd |  |  |  |
| Brod[9] | 1988 | RP | 75 | M | headache, personnality change, nystagmus | ME |  |  |  | 3-90 (Ly / PM) |  |  | Cs | yes | yes | Meningeal biopsy: lymphocytic meningitis |
| Brod[9] | 1988 | RP | 62 | F | headache | pachyM | CT: pachymeningitis |  |  | 0-6 (Ly) |  |  | Cs, MTX | ? |  |  |
| Morita[10] | 1988 | RP | 41 | M | pseudo-DRPLA: myoclonus, chorea, ataxia | unclear | CT: midbrain/mesencephalon atrophy ? |  |  | 1 | 0.7 | high IgG |  |  |  |  |
| Stewart[11] | 1988 | RP | 52 | M | visual acuity, headache, then delirium, then seizures | ME | nd |  |  | 200 (70 PM) | 1.77 |  | Cs | no | yes (multiple) | autopsy: vasculitis small veins+arteries, with thrombosis; perivascular cuffing; 'punched out' cortical scars |
| Schindzielorz[12] | 1991 | RP | 64 | M | headache | M | nd |  |  | 440 (80) to 550 (70PM) | 0.45 |  | Cs, Cyc, Abttt | yes | yes (same) | strongyloides meningitis at relapse ? |
| Strobel[13] | 1992 | RP | 51 | F | none | aneurysm | basilar artery aneurysm |  |  |  |  |  | Cs, Aza | yes (general), aneurysm unchanged |  |  |
| Wasserfallen[14] | 1992 | RP | 73 | F | Horner, facial paresis, neck stiffness | rhombencephalitis | nd? |  |  | 950 (68 PM) | 1 |  | Cs, Cyc | yes | no |  |
| Bouton[15] | 1994 | RP | 73 | F | stroke, then 2 months later confusion and chondritis | stroke | topography unclear |  |  |  |  |  | Cs | yes | no | arteriography: normal |
| Hanslik[16] | 1994 | RP | 70 | F | headache, confusion, | ME | multifocal WM, periventricular, subcortical and capsular lesions |  |  | 22 (50) |  |  | Cs, dapsone | yes | no |  |
| Berg[17] | 1995 | RP | 60 | M | headache, papilledema, ataxia, SNHL | M | 1st attack: CT: normal, 2nd attack: MRI: pachyM, hydrocephalus ? |  |  | 140 (71) to 1990 (86 PMN) | 1.4 - 1.9 |  | spontaneous remission, then CS, Aza | yes (same) | yes, general | meningeal biopsy: mild inflammation (PMN, Ly) |
| Ragnaud[18] | 1996 | RP | 70 | M | Aseptic meningitis (6 times) during flares | M | nd | nd | nd | 1700 to 4000 (73 to 98 PM) | 1.7 to 4 | hypoglyco 1 to 2.1 mmol/L | Cs | yes | yes (x6) |  |
| Kothare[19] | 1998 | RP | 68 | M | partial seizure | ME | multifocal frontal WM | fronto-parietal LM enh. |  | 27 (30) | 0.59 |  | Cs | yes | no | angiography: normal. Brain biopsy: necrosis, mononuclear meningeal infiltration, no vasculitis |
| Ramos[20] | 1999 | RP | 73 | M | headache | M | normal | none? | yes | 130 (90) | 0.89 |  | Cs | yes | no |  |
| Dreher[21] | 2000 | RP | 75 | M | Tremor | ME | multifocal confluent patchy NS WM lesions ("vasculitis") | none? |  |  |  |  | Cs | yes (non neuro?) | no (not pertinent) |  |
| Coumbaras[22] | 2001 | RP | 50 | M | Headache during flare | unclear |  |  |  |  |  |  |  |  |  | Brain angio.: CAA aneurysm |
| Brucki[23] | 2001 | RP | 55 | M | Recurring fever and confusion | Encephalopathy | nd |  | yes | 277 (78 PN), 14 (89), 122 (78 PN) | normal |  | Cs | yes | yes |  |
| Sost[24] | 2001 | RP | 72 | F | dementia, hallucination, headache | ME | hydrocephaly, |  | yes | 56 (64 PM) | 0.39 |  | Cs, Cyc | yes | yes (same) |  |
| Yamazaki[25] | 2001 | RP | 58 | F | bilateral internal arotid stenosis, headache | vasculitis |  |  |  |  |  |  | Cs, AZA | yes |  | MRA: bilateral arterial moderate stenosis: regression after Cs |
| Elmaci[26] | 2021 | RP | 37 | F | hand numbness, cervical pain | pachyM (no-ANCA, IgG4+) | extramedullary mass C2-T3 |  |  |  |  |  | surgery | yes |  | pathology: IgG4-related |
| Hosford[27] | 2003 | RP | 61 | M | progressive cognitve impairment for 1 year, transient dysarthria | ME | multiple foci WM and basal ganglia |  | yes | high | high |  | Cs | no | yes (scleritis) |  |
| Topalkara[28] | 2003 | RP | 44 | M | Wallenberg | stroke | bulbar stroke |  |  |  |  |  | Cs, AZA | yes | no | angio: basilar A irregular, PICA occlusion: normalized at month 2 |
| Fujiki[29] | 2004 | RP | 45 | M | Subacute confusion | E | Bilateral cortical temporal and insular lesions | nd | yes | 8 (94) | 0.86 | IgG index | Cs | yes |  | Brain biopsy: perivascular ly cuffing |
| Fujiki[29] | 2004 | RP | 62 | M | Subacute dementia | E | Bilateral temporal and WM lesions | nd | yes | 24 (83) | 0.46 | IgG index | Cs | yes |  | Brain biopsy: perivascular ly cuffing |
| Gertner[30] | 2004 | RP (Magic) | 38 | M | subacute dementia | inflammatory lesion (Behçet type) | unilateral lesion (mesenceph-dienceph.) |  |  |  |  |  | Cs, Aza | yes |  |  |
| Ohta[31] | 2004 | RP | 57 | M | SNHL, then fever headache vertigo, then dementia | ME | normal, then bitemporal, caudate, WM lesions | normal then paraventricular WM |  | 68-119 (85) | 0.57-0.86 | high IgG index, no OCB |  | yes | no | MRA normal |
| Yang[32] | 2004 | RP | 49 | M | confusion then headache and ataxia | ME | multifocal WM, temporal, caudate, striatum and thalamus |  |  | 145 (58) | 0.87 |  | Cs, then HCQ, AZA | yes | yes |  |
| Ota[33] | 2005 | RP | 57 | F | delirium, then SNHL, then seizure | ME | nd | PM+LM enh. | yes | 196-1056 (63 Ly to 84 PM) | 0.89 | high IgG index | Cs | yes partial |  |  |
| Head[34] | 2006 | RP | 71 | M | Slow dementia (Lewy body like) | ME |  |  |  |  |  |  |  |  |  | Autopsy: leptomeningitis, vasculitis |
| Hsu[35] | 2006 | RP | 71 | F | headache, confusion | M | normal, then stroke, MCA arteritis |  |  | 110 (100) | 1.16 |  | CS | yes | yes (stroke) |  |
| Irani[36] | 2006 | RP | 65 | M | headache, subacute confusion | ME | Bilateral temporal and multifocal WM lesions | none | yes | 65 | 0.78 |  | Cs, Cyc | yes (clinic), MRI improv. |  |  |
| Sato[37] | 2006 | RP | 51 | M | headache, nasal congestion | pachyM | PM frontal invading the skull | idem |  |  |  |  | Surgery, Rxttt | yes | yes (general) | surgery: plasma cell granuloma |
| Yan[38] | 2006 | RP | 53 | M | dementia | E | bitemporal lesions |  |  | normal |  |  |  | no treatment? | death | Autopsy: leptomeningitis, perivascular infl. |
| Choi[39] | 2007 | RP | 54 | F | headache, then memory troubless | ME | bitemporal, diffuse WM and striatum | basal ganglia, WM and subcortical foci | yes | 290 (93 PM) | 1.6 |  | Cs then Aza | yes | yes (chondritis, SNHL) |  |
| Hirunwiwatkul [40] | 2007 | RP | 78 | F | recurrent painless unilateral optic neuritis | optic neuritis |  | orbital periostitis |  |  |  |  | Cs | yes | yes (same) |  |
| Kao | 2007 | RP | 40 | M | Headache, confusion | ME | Bilateral cortical temporal, insular, frontal, striatal lesions | nd |  | 45 (62 PM) to 1500 (83 PM) | 0.85 |  | Cs | yes |  |  |
| Baaj[41] | 2008 | RP | 48 | F | headache, limb paresthesia | pachyM (no-ANCA) |  | unilateral irregular pachyM |  | nd |  |  | Cs, MTX, Cyc, etanercept (monottt at neurological onset) > surgery |  |  | biopsy: granulomas, giant cells, extensive necrosis (provisional diag: Wegener although no renal/pulmonary/ANCA) |
| Ertens-Lyon[42] | 2008 | RP | 51 | M | Ataxia, rapid dementia, myoclonic jerks | ME | Extensive NS WM lesions | none ? |  | 39 (65) | 0.89 | IgG index | Cs, Cyc | no | no | FDG: low cortical. Brain biopsy: NS inflammation, no vasculitis. Angiography: normal |
| Ertens-Lyon[42] | 2008 | RP | 68 | M | Rapid dementia | E | Edema right medial tempora, then bilateral caudate heads | caudate heads | yes | 4 (44) | 0.49 |  | Cs | yes |  | Autopsy: meningeal inflammation, microglial activation |
| Fujioka[43] | 2008 | RP | 66 | F | Subacute dementia, parkinsonism, akinetic mutism | E | multifocal WM and striatal lesions | none |  | 90 | 1.47 | IgG index | Cs | yes (clinic), MRI improv. | no |  |
| Kuwabara[44] | 2008 | RP | 61 | M | Seizures, headache, subacute cognitive decline, hallucinations | ME | cortical edema R temporal lobe | enh. temporal gyri |  | 312 (99) | nd |  | Cs | yes | nd |  |
| Andrade-Ramos[45] | 2009 | RP | 67 | M | Cognitive, parkinsonism, myoclonus | E | multifocal WM lesions, large tempropariet. cortical lesions (diff+) | nd | yes | nd |  |  | Cs, Cyc | minor | death |  |
| Andrade-Ramos[45] | 2009 | RP | 35 | M | Headache, papilledema | E | posterior striatum and WM, LM | LM |  | normal |  |  | Cs | yes | nd |  |
| Imamura[46] | 2009 | RP | 76 | F | subacute dementia, consciousness | ME | bitemporal edema and progressive diffuse WM | none | yes | 73 (100) | 1.2 | IgG index | Cs then MTX, CycA | yes | yes (same) | Brain biopsy: Ly, plasma cells, PMN, Eosino in meninges and perivascular cuffing. No vasculitis. Autopsy: diffuse demyelination around blood vessels, no vasculitis |
| Kashihara[47] | 2009 | RP | 62 | M | headache, subacute cognitive decline, hallucinations, seizures, past aspetic meningitis | Limbic E | Bilateral temporal lesions (mild) | none | yes | 39 (59) |  | GLUR2 (NR2B) | Cs, IVIG, tacro | no |  |  |
| Kumar[48] | 2009 | RP (Magic) | 29 | M | Cognitive? | Limbic E | Bilateral temporal lesions | none |  | 32 (Ly) | 0.60 |  | Cs, Aza, adalimumab | yes | nd |  |
| Pamuk[49] | 2009 | RP | 57 | F | Trigeminal neuralgia during flare | CN (neuralgia) | normal |  |  |  |  |  | Cs, MTX, etanercept | yes | no |  |
| Swen[50] | 2009 | RP | 58 | M | subacute dementia, visual hallucinations | E | normal (too early?) |  |  | nd |  |  | Cs | yes | yes (chondritis) |  |
| Yaguchi[51] | 2009 | RP | 56 | F | headache, VI CN | M | normal |  |  | 640 (94 PM) | 1.1 | no | Cs | yes | no |  |
| Sampaio[52] | 2010 | RP | 70 | M | confusion, hallucination | ME | periventricular WM | periventricular enh. |  | 38 (Ly) | 0.62 |  | Cs | yes |  |  |
| Canas[53] | 2011 | RP | 50 | F | headache, facial hypesthesia | pachyM, CN | meningeal lesion | thick PM posterior fossa and tentorium cerebelli | no | nd | nd |  | CS | yes | yes | PR3-ANCA+ |
| Canas[53] | 2011 | RP | 48 | F | headache, diplopia | pachyM, CN | pachyM | diffuse PM convexity |  | nd | nd |  | Cs, Cyc | yes |  | PR3-ANCA+ |
| Canas[53] | 2011 | RP | 50 | F | headache, "multiple cranial palsy" | pachyM ? | pachyM ? | posterior fossa pachyM ? |  | nd | nd |  | Cs, Cyc, RTX | yes |  | PR3-ANCA+ |
| Choi[54] | 2011 | RP | 68 | F | Acute confusion aphasia | E | WM anterior horns | diffuse LM |  | 33 (67) | 0.55 |  | Cs, MTX | yes | no |  |
| Garcia-Egido[55] | 2011 | RP | 57 | M | Confusion, fever | ME | multifocal WM lesions | patchy WM ? |  | 700 (98) | 0.75 |  | Cs, Cyc, MTX, inflix | yes | yes, 7 ME without negative CSF |  |
| Roux[56] | 2011 | RP | 57 | M | ataxia, cochlear and vestibular signs | SNHL |  |  |  |  |  |  | Cs | yes | no |  |
| Storey[57] | 2011 | RP | 73 | M | headache, seizure, confusion, mild ataxia, myoclonue | ME | bitemporal lesions |  | yes | 89 (Ly) | 0.66 |  | Cs, PLEX, IVIG | no | death | autopsy: meningitis, Ly cuffing and microglial nodules |
| Wang[58] | 2011 | RP | 54 | M | headache, memory loss, hallucinations | ME | diffuse WM |  | yes | 0 - 800 (95) | 0.6 |  | Cs, AZA | yes | yes, meningitis |  |
| Wang[58] | 2011 | RP | 44 | M | Amnesia for 20 days | ME | Left hppocampus, right temporal WM |  |  | 190 (90PM) | 0.57-0.67 |  | Cs, AZA | yes | yes, meningitis |  |
| Wang[58] | 2011 | RP | 52 | M | mental changes for 12 months, parkinsonism | ME | ventricular enlargement |  |  | 230 (70) | 0.5 - 1 |  | Cs, IVIG | yes | yes, meningitis |  |
| Wang[58] | 2011 | RP | 44 | F | Brudzinski | M | normal |  |  | 70 (60) | 0.71 |  | Cs, AZA | yes | yes, chondritis |  |
| Defer[59] | 2012 | RP | 67 | M | Parkinsonism and frontal signs over 2 months | ME | multifocal WM and striatal lesions | patchy striatal and WM | no |  |  |  | Cs | yes | yes |  |
| Prinz[60] | 2012 | RP | 63 | M | headache, confusion | ME | caudate, multifocal WM and a few cortical | basal ganglia and subcortical foci |  | 33 - 106 (Ly) | 0.76 - 0.93 |  | Cs, Aza | yes | yes (chondritis) |  |
| Ducci[61] | 2017 | RP | 69 | M | Ataxia, vertigo, tinnitus, downward nystagmus, confusion, paraparesis | ME | mild thickening of the dura |  |  | inflammation |  |  | Cs | yes |  |  |
| Kiliç Coban[62] | 2013 | RP | 43 | F | stroke (MCA), possible cardiac insufficiency | stroke |  |  |  |  |  |  |  |  |  |  |
| Mattiassich[63] | 2013 | RP | 49 | M | no CNS sign | stroke ? Vasculitis? | old lacunar lesions lenticular, WM | nd |  | nd |  |  |  |  |  |  |
| Moemoe[64] | 2013 | RP | 54 | M | subacute dementia, headache, then hallucinations | ME | normal (too early?) |  |  | 59 | 0.62 |  | Cs, Aza | yes | yes (chondritis) |  |
| Hatti[65] | 2014 | RP | 56 | M | Encephalitis, headache | ME | multifocal WM and striatal lesions | nd |  | 204 (82) | 1.62 |  | Cs, Cyc | yes |  | Brain biopsy: arachnoiditis, gliosis, granuloma |
| Kondo[66] | 2014 | RP | 58 | M | Subacute dementia | Limbic E | none | none | yes | 33 (99) | 0.92 | GLUR2 (NR2B) | Cs, infliximab | no |  |  |
| Nishigushi[67] | 2014 | RP | 61 | M | amnesia, confusion | limbic E | bitemporal edema |  |  | 340 (90 PM) | 0.89 |  | Cs | yes | yes (chondritis) |  |
| Niwa[68] | 2014 | RP | 59 | M | typical unilateral parkinsonism with right tremor for 5 years before | ME | cortical temporal, insula, striatal WM., mainly right |  |  | 25 (100) | 0.87 |  | Cs | yes | yes, same, death | Autopsy: perivascular cuffing instriatum, and typical LBD |
| Baba[69] | 2015 | RP | 72 | M | Drowsiness over 2 days, seizure | E | diffuse WM, callosal edematous, cerebellar peduncle | none | yes (callosal+), partial impr. WM | 781 (87 PN) | 5.8 |  | Cs, Cyc | yes (clinic) | no |  |
| Jeon [70] | 2015 | RP | 48 | F | subacute headache, vomiting | ME, vasculitis (stroke) | cerebellar and striatal lesions. Occlusion MCA (arteritis) | nd | nd | 71 (58) | 0.86 |  | Cs | no | death |  |
| Jeon [70] | 2015 | RP | 56 | M | confusion, hallucination | Limbic E | normal | nd | nd | 25 (60) | 0.62 |  | Cs | yes | no |  |
| Jeon [70] | 2015 | RP | 48 | M | cognitive for 2 months | Limbic E | normal | nd | yes | 18 (74) | 0.32 |  | Cs, MTX, Aza | no | lost FU | Brain FDG-Pet: decreased temporal , cerebellum |
| Hwang[71] | 2015 | RP | 53 | M | Cognitive, parkinsonism, myoclonus | ME | multifocal WM and cortical lesions | multiple nodular WM, cortex and LM | nd | 38 (76) | 1.08 |  | Cs | yes | nd |  |
| Karapanayiotides  [72] | 2015 | RP | 52 | M | Transient dysarthria | thrombosis | Floatting carotid thrombus (+atheroma) |  |  |  |  |  | CS, Aza | yes | no |  |
| Kilic Coban[62] | 2015 | RP | 44 | F | stroke during a flare | stroke |  |  |  |  |  |  |  |  |  |  |
| Nakamura[73] | 2015 | RP | 81 | F | dementia | ME, pachyM | multifocal WM lesions | confluent frontal LM, falx |  | 12 (100) | 0.65 | OCB+ | Cs | yes | nd | frontal meningeal diff. restricted |
| Nara[74] | 2015 | RP | 39 | M | headache, confusion | ME | multifocal WM lesions, and cerebellar peduncles | nd | yes | 19 (100) | 0.71 |  | Cs | yes | no |  |
| Almackenzie[75] | 2016 | RP | 37 | M | confusion, transient blindness over 2 months | ME | multifocal WM, striatal lesions | multifocal minute WM, cortex, LM |  | 444 (59) | 0.71 |  | Cs, Cyc, MMF | yes | no | TOF: "mild vasculopathic changes". Biopsy: perivascular infiltrate, no vasculitis |
| Chernyak[76] | 2016 | RP | 39 | M | headache, dizziness | M |  |  |  | 400 (84) | 2.02 |  | CS, MTX | yes | yes (same), death (unrelated) |  |
| Chernyak[76] | 2016 | RP | 64 | M | diplopia, hoarsness of the voice (unilat. VII and XII ?), ocular signs | stroke ? E ? | multifocal WM, strokes in basal ganglia ? |  |  | nd |  |  | Cs | yes | no |  |
| Chernyak[76] | 2016 | RP | 65 | M | one month subacute optic neuropathy ("ischemic") | E ? | multifocal WM lesions? |  |  | nd |  |  | Cs | incomplete |  |  |
| Dion[77] | 2016 | RP |  |  | lymphocytic meningitis | M |  |  |  |  |  |  |  |  |  |  |
| Dion[77] | 2016 | RP |  |  | cerebral vasculitis | brain vasculitis |  |  |  |  |  |  |  |  |  |  |
| Dion[77] | 2016 | RP |  |  | cranial nerve lesions | CN |  |  |  |  |  |  |  |  |  |  |
| Dion[77] | 2016 | RP |  |  | polyneuropathy | PNP |  |  |  |  |  |  |  |  |  |  |
| Dion[77] | 2016 | RP |  |  | Mononeuropathy multiplex | PNP |  |  |  |  |  |  |  |  |  |  |
| Ellis | 2016 | RP | 51 | M | cognitive change over a year | E | Unclear. medial temporal atrophy ? |  | yes | normal |  |  | MTX | stable | no |  |
| Ellis [78]; Mbizvo | 2016 | RP | 65 | M | Memory, then TIA, then confusion and fever over 2 months | ME | multifocal WM lesions (figure 2: cortical ?) | none | no | 44 (100) | 0.6 |  | Cs, Cyc | yes | no | CT angiography: no vasculitis. Brain biopsy: parenchyma: scattered foci of vasculitis; multinucleated giant cells, Tcells, PMN, eosino; meninges: inflammatory infiltrates |
| Eun[79] | 2016 | RP | 60 | M | Left CN III, IV, VI, paresthesia V | pseudotumor orbital | pseudotumoe orbital apex and Meckel's cave | pachymening. cavernous enh. | no | 0 | 0.23 |  | Cs | yes | no |  |
| Kamboj[80] | 2016 | RP | 65 | M | VII, shoulder pain, U SNHL | M /pseudotumor ? | spinal meningeal thickening | enh. VII-VIII nerves | nd | nd | nd |  | CS, Azacytidine | yes | no |  |
| Kawabe[81] | 2016 | RP | 67 | M | amnesia for 2 months | ME | medial temporal lobes and insular |  |  | 47 (Ly) | 0.67 |  | Cs | yes | no |  |
| Lin[82] | 2016 | RP |  |  | aseptic meningitis | M |  |  |  |  |  |  |  |  |  |  |
| Lin[82] | 2016 | RP |  |  | raised intracranial pressure | unclear |  |  |  |  |  |  |  |  |  |  |
| Lin[82] | 2016 | RP |  |  | brain infarction | stroke |  |  |  |  |  |  |  |  |  |  |
| Lin[82] | 2016 | RP |  |  | auricular and occipital neuritis | CN (neuralgia) |  |  |  |  |  |  |  |  |  |  |
| Lin[82] | 2016 | RP |  |  | epilepsy | seizure (isolated) |  |  |  |  |  |  |  |  |  |  |
| Lin[82] | 2016 | RP |  |  | slurred speech and memory | E |  |  |  |  |  |  |  |  |  |  |
| Lin[82] | 2016 | RP |  |  | Cerebral vein thrombosis | stroke |  |  |  |  |  |  |  |  |  |  |
| Lin[82] | 2016 | RP |  |  | ataxia | unclear |  |  |  |  |  |  |  |  |  |  |
| Liu[83] | 2016 | RP | 60 | F | confusion, headache | E | minute striatal lacunes (?) | nd |  | normal |  |  | Cs, TCZ, then MTX then Cyc | yes |  |  |
| Maillet[84] | 2016 | RP | 38 | M | confusion, seizure, meningeal signs | ME, pachyM |  | diffuse LM enh. |  | 150 (66) | 0.39 |  | Cs, Cyc, AZA | yes | no | arteriography: normal |
| Simabukuro[85] | 2016 | RP | 43 | M | headache, seizures, amnesia | ME | temporal edema |  | yes | 105 (77) | high |  | Cs | yes |  |  |
| Ushiyama[86] | 2016 | RP | 80 | M | headache, SNHL, facial sensory | pachyM (no-ANCA) |  | diffuse pachyM |  | 57 (100) | 0.78 | IgG index | Cs | yes | no |  |
| Ahn[87] | 2017 | RP | 33 | M | decrease mentation, dysarthria, sleepiness, headache | E | WM and cortical HS FLAIR | multiple nodular | yes, progressive | 20 (58) | 0.64 | IgG index, OCB- | Cs, MMF, CyC | yes (except atrophy) | yes, same |  |
| Dal-Pra Ducci[61] | 2017 | RP | 69 | M | paraparesia, vertigo, tinnitus, ataxia, confusion | ME | nd | diffuse LM on cervical cord and |  | 71 (93) | 0.69 |  | Cs, MTX | partial | no |  |
| Ismail[88] | 2017 | RP | 57 | F | V1, XII | pseudotumor (CN) | bilateral skull base, lumbosacral, orbital pseudotumor | yes | nd | normal | normal | OCB+ | Cs | yes | nd |  |
| Le Marec[89] | 2017 | RP | 34 | F | meningitis, then diplopia (VI x2) | M | nd | multifocal LM (including premesenceph.) |  | 150 (Ly) | 1.19 |  | MTX, infliximab | yes | nd |  |
| Ma[90] | 2017 | RP | 62 | F | hydrocephalus (no data) | hydrocephalus |  |  |  |  |  |  |  |  |  |  |
| Shen[91] | 2017 | RP | 58 | F | headache | M | pachymeningitis (dubious?) |  |  | 150 (58 PM) | 0.28 |  | Cs, MTX then Cyc, cycloA | yes (CycloA) | yes, SNHL, chondritis |  |
| Tsai[92] | 2017 | RP | 44 | M | Headache, seizures, confusion, hallucinations | ME | multifocal WM lesions | nd |  | nd |  |  | Cs, Cyc | yes | no | normal TOF |
| Zhou[93] | 2017 | RP | 26 | M | headache, mental disorder | ME | nd |  |  | 22 | 0.28 |  | Cs | yes |  |  |
| Zhou[93] | 2017 | RP | 48 | F | headache, dizziness, bulbar, paralysis | ME | nd |  |  | 40 (60) | 0.31 |  | Cs | yes |  |  |
| Zhou[93] | 2017 | RP | 46 | M | headache, seizure, aphasia, mental disorder | ME | nd |  |  | 146 (59) | 0.85 |  | Cs | yes |  |  |
| Zhou[93] | 2017 | RP | 41 | M | headache, limb weakness, mental disorder | ME | nd |  |  | 6 | 0.6 |  | Cs | yes |  |  |
| Zhou[93] | 2017 | RP | 46 | M | headache, SNHL | ME | nd |  |  | 30 (100) | 0.3 |  | Cs | yes |  |  |
| Zhou[93] | 2017 | RP | 51 | M | headache, dizziness, bulbar paralysis, vision, facial palsy | ME | nd |  |  | 15 | 0.41 |  | Cs | yes |  |  |
| Zhou[93] | 2017 | RP | 38 | M | headache, SNHL, bulbar paralysis, facial | ME | nd |  |  | 22 (23) | 0.7 |  | Cs | yes |  |  |
| Cao[94] | 2018 | RP | 64 | M | Headache for 4 months, paraparesia, cognitive | ME | Multifocal WM ? | LM sulci | yes, and hydrocephaly | 520 (40) | 0.77 | yes | Cs, MTX | yes | no |  |
| Cao[94] | 2018 | RP | 64 | M | headache, confusion | ME | WM, hydrocephalus | LM enh. Occipitotemporal | yes | 0 to 260 (80) | 0.27 to 0.77 | yes | Cs, MTX | yes | no |  |
| Al-Tabaa[95] | 2018 | RP | 25 | M | Visual hallucinations, depression over 6 months | E | Diffuse patchy WM/bicaudate HS FLAIR, worsened diffuse major irregular WM, cortical | nd | yes, and hydrocephaly | 21 (80), worsened 468 (15 Ly, 68 PN) |  |  | Cs,n Cyc | yes (clinic), no (MRI), than severe relapse | yes (status epilepticus) | Dural biopsy: meningeal inflammation, Ly, plasmocytes. Perivasqcular histiocytes and Ly without vasculitis |
| Shashikala[96] | 2018 | RP | 52 | M | headache, facial nerve | M | normal (done after clinical improvement by Cs) |  |  | 16 | normal |  | Cs, Cyc | yes |  |  |
| Zhu[97] | 2018 | RP | 66 | M | partial seizure, ataxia, dementia | ME | multifocal WM and cortical, striatal lesions | nd | yes | 8 | 0.79 | yes | Cs, IVIG | yes | yes (same) |  |
| Miyano[98] | 2019 | RP | 71 | F | incidental abnormal brain MRI during exploration of scleritis | ME | multifocal WM and insular | focal enh. | no | nd |  |  | Cs | yes | nd |  |
| Anada[99] | 2020 | RP | 65 | M | Drowsiness, urinary retention | ME | abnormal basal ganglia, and spinal cord normal | nd | nd | nd |  |  | Cs (worsening > Cs, Cyc) | yes | no | FDG uptake: spinal +(pharynx, nose) |
| Angkodjodjo[100] | 2020 | RP | 66 | M | Confusion over 6 weeks (MMS 11/30) | Limbic E | nd (PM) | nd | nd | 48 (74) | >2 |  | Cs, Cyc | yes clinic+PET | no | FDG uptake: Left hippocampal (ear) ±striatum ? |
| Bravo-Ruiz[101] | 2020 | RP | 43 | M | Confabulations, seizure, transient aphasia | E | multifocal WM, temporal, striatal and thalamic lesions | none | yes | 0 | 0.58 |  | Cs, Cyc, IVIG | yes | no |  |
| Chaucer[102] | 2020 | RP | 41 | F | basilar SHA | Sub arachnoid haemorrhage |  |  |  |  |  |  |  |  |  |  |
| Farhat[103] | 2020 | RP | 44 | M | pachymeningitis | pachyM | normal ? | minute fronto-parietal PM enh; |  | nd |  |  | Cs, TCZ | yes (unclear concerning pachyM) | no |  |
| Ikeda[104] | 2020 | RP | 23 | F | Coma, seizure | E (PRES) | PRES | none |  | 2 (100) | 0.41 |  |  | yes | yes (general) |  |
| Lin[105] | 2020 | RP / GFAP | 38 | M | Repeated abnormal behavior then aphasia, diplopia, tremor, limb weakness | ME | well-limited round high signal bilateral hippocampus, basal ganglia, periventricular and centrum semi ovale WM | Multiple focal enh. |  | 15 |  |  | Cs, IVIG, MMF. | yes | yes (same and severe) | GFAP(+) |
| Matsuzono[106] | 2020 | RP | 80 | M | dementia | ME | typical ABRA (confirmed by PIB PET) | diffuse LM enh. |  | 8 | nd | nd | Cs | yes | no |  |
| Cao[107] | 2021 | RP |  |  | Meningoencephalitis | ME |  |  |  | 90 | 0.51 |  |  |  |  |  |
| Cao[107] | 2021 | RP |  |  | Meningoencephalitis | ME |  |  |  | 500 | 1.81 |  |  |  |  |  |
| Cao[107] | 2021 | RP |  |  | Meningoencephalitis | ME |  |  |  | 96 | 0.99 |  |  |  |  |  |
| Cao[107] | 2021 | RP |  |  | Aseptic meningitis oculomotor nerves+involvement of bilateral | M, CN |  |  |  | 2 | 0.97 |  |  |  |  |  |
| Cao[107] | 2021 | RP |  |  | Limbic encephalitis+myelitis | ME, SC |  |  |  | 12 | 0.47 |  |  |  |  |  |
| Cao[107] | 2021 | RP |  |  | Encephalitis+cranial nerves involvement ofVII, IX,X,and XII | ME, CN |  |  |  | 202 | 0.77 |  |  |  |  |  |
| Cao[107] | 2021 | RP |  |  | Meningoencephalitis+medullary syndrome+nerves incomplete dorsolateral injury of V and VII cranial | ME, CN |  |  |  | 30 | 0.66 |  |  |  |  |  |
| Cao[107] | 2021 | RP |  |  | Aseptic meningitis cranial nerves+ involvement of III and VI | M, CN |  |  |  | 70 | 0.737 |  |  |  |  |  |
| Cao[107] | 2021 | RP |  |  | Acute encephalomyelitis | ME, SC |  |  |  | 8 | 0.58 |  |  |  |  |  |
| Cao[107] | 2021 | RP |  |  | Meningoencephalitis | ME |  |  |  | 4 | 0.619 |  |  |  |  |  |
| Cao[107] | 2021 | RP |  |  | Meningoencephalitis | ME |  |  |  | 42 | 0.75 |  |  |  |  |  |
| Cao[107] | 2021 | RP |  |  | Encephalitis | E |  |  |  | 0 | 0.49 |  |  |  |  |  |
| Cao[107] | 2021 | RP |  |  | Aseptic meningitis | M |  |  |  | 0 | 0.5 |  |  |  |  |  |
| Cao[107] | 2021 | RP |  |  | Limbic encephalitis | Limbic E |  |  |  | 0 | 0.55 |  |  |  |  |  |
| Cao[107] | 2021 | RP |  |  | Aseptic meningitis | M |  |  |  | 713 | 0.61 |  |  |  |  |  |
| Cao[107] | 2021 | RP |  |  | Limbic encephalitis | Limbic E |  |  |  | 12 | 0.49 |  |  |  |  |  |
| Cao[107] | 2021 | RP |  |  | Aseptic meningitis | M |  |  |  | 56 | 0.57 |  |  |  |  |  |
| Cao[107] | 2021 | RP |  |  | Limbic encephalitis | Limbic E |  |  |  | 1 | 0.24 |  |  |  |  |  |
| Cao[107] | 2021 | RP |  |  | Manifestation similar with intracranial hypertension | unclear |  |  |  | 0 | 0.27 |  |  |  |  |  |
| Cao[107] | 2021 | RP |  |  | Encephalitis | E |  |  |  | 80 | 0.86 |  |  |  |  |  |
| Cao[107] | 2021 | RP |  |  | Limbic encephalitis | Limbic E |  |  |  | 0 | 0.48 |  |  |  |  |  |
| Cao[107] | 2021 | RP |  |  | Aseptic meningitis | M |  |  |  | 12 | 0.28 |  |  |  |  |  |
| Cao[107] | 2021 | RP |  |  | Encephalitis | E |  |  |  | 3 | 0.56 |  |  |  |  |  |
| Cao[107] | 2021 | RP |  |  | Optic (peri?)neuritis | CN |  |  |  |  |  |  |  |  |  |  |
| Cao[107] | 2021 | RP |  |  | Optic (peri?)neuritis | CN |  |  |  |  |  |  |  |  |  |  |
| Contreras[108] | 2021 | RP | 22 | F | Headache, chorioretinitis, cone infl. | M | normal | diffuse LM, including cerebellar |  | 180 (96) | 0.46 |  | Cs, IS | yes | no |  |
| Cordts[109] | 2021 | VEXAS | 60 | M | acute myofasciitis with capillary leak syndrome | myositis |  |  |  |  |  |  | CS, Azacytidine | yes | no |  |
| Fan[110] | 2021 | RP | 69 | M | headache, side weakness, confusion | stroke, vasculitis, leptoM | multifocal strokes. | diffuse LM enh. Neuraxis |  | high |  |  | Cs | yes |  | MRI angio: vasculitis (wall enh. Irregular arteries) |
| Hutto[111] | 2021 | RP | 48 | F | headache | pseudotumor (focal pachyM) | PM (en plaque) | frontotemporal PM (en plaque) |  | 5 (69) | 0.31 | no ITS | Cs, adalimumab | yes |  |  |
| Matsumoto[112] | 2021 | RP | 61 | M | headache, confusion | ME | global edema, temporal cortex ? |  |  | 189 (52) | high | IgG index, OCB- | Cs, MTX | yes | no |  |
| Obiorah[113] | 2021 | VEXAS |  |  | minor stroke (no detail) | stroke |  |  |  |  |  |  |  |  |  | no cardiac or atherosclerotic lesion |
| Van der Made[114] | 2021 | VEXAS | 56 | M | headache | headache | nd |  |  | nd |  |  |  |  |  |  |
| Van der Made[114] | 2021 | VEXAS | 76 | M | headache | headache | normal |  |  | normal |  |  |  |  |  |  |
| Van der Made[114] | 2021 | VEXAS | 55 | M | stroke during a flare | stroke |  |  |  |  |  |  |  |  |  |  |
| Van der Made[114] | 2021 | VEXAS | 76 | M | Polyneuropathy (forearms) | PNP |  |  |  |  |  |  |  |  |  |  |
| Van der Made[114] | 2021 | VEXAS | 78 | M | Axonal polyneuropathy | PNP |  |  |  |  |  |  |  |  |  |  |
| Van der Made[114] | 2021 | VEXAS | 74 | M | Minor stroke during a flare | stroke | no brain vasculitis |  |  |  |  |  |  |  |  |  |
| Van der Made[114] | 2021 | VEXAS | 47 | M | Aseptic meningitis | M |  |  |  |  |  |  |  |  |  |  |
| Vera[115] | 2021 | RP | 32 | F | headache, confusion, hallucinations | ME | caudate, multifocal WM and a few cortical | punctate WM (some cortical) | no? | 85 (95 PM) | 0.96 |  | Cs, Cyc | yes | no |  |
| Xu[116] | 2021 | RP | 41 | M | seizure (then episcleritis, chondritis at d3) | ME | focal deep WM | LM sulcus (FLAIR), patchWM and cortex (T1) |  | 17 (92) | normal |  | Cs | yes | nd |  |
| Bert-Marcaz[117] | 2022 | VEXAS | 74 | M | acute-onset CIDP | GBS | nd | nd |  | 2 | 0.8 |  | IVIG, Cs | yes | yes (general) |  |
| Collantes-Rodriguez[118] | 2022 | VEXAS | 60 | M | Acute demyelinating polyneuropathy | PNP |  |  |  |  |  |  | multiple IS | nd | nd |  |
| Escoda[119] | 2022 | VEXAS | 69 | M | Lewis Sumner | PNP |  |  |  | Normal | Normal |  | IVIG, Azacytidine | yes | yes, same |  |
| Guerreo-Bermudez[120] | 2022 | VEXAS | 72 | M | LL myositis | myositis |  |  |  |  |  |  | Cs, MTX | yes | yes |  |
| Holmes[121] | 2022 | VEXAS | 70 | M | Headache, aphasia, trigeminal neuralgia | Brain pseudotumor, CN | nd | nd |  |  |  |  | Cs | nd |  | Brain biopsy: lymphocytoplasmacytic infiltrate |
| Lee[122] | 2022 | RP | 52 | M | gait, arthric, cognitive disturbance | ME, vasculitis (stroke) | meningeal lesions, punctate WM stroke | multifocal LM |  | 56 (73) |  |  | Cs | yes |  | normal TOF |
| Rachdi[123] | 2022 | RP | 52 | M | headache, papilloedema | pachyM (no-ANCA) |  | left convexity pachyM |  | 11 (88) | normal | IgG index, OCB+ | Cs | yes | no |  |
| Topilow[124] | 2022 | VEXAS | 57 | M | myofasciitis with normal CK, orbital myositis | myositis | fasciitis |  |  |  |  |  |  |  |  |  |
| Yokota[125] | 2022 | RP | 49 | M | cognitive impairment for 6 months | E | Bilateral temporal, multifocal WM, striatal, cortical lesions | multifocal WM and cortical | yes | 1 | 0.54 | no | Cs, Cyc, AZA, MTX | yes | no |  |
| Yoon[126] | 2022 | VEXAS | 66 | M | multifocal myositis | myositis |  |  |  |  |  |  |  |  |  |  |
| Belicard[127] | 2023 | VEXAS | 70 | M | LL bilateral progr. Sensory, hyperesthesia, proprioception, then delirium (post-cardiac arrest) | PNP | normal |  |  | 0 | 0.6 |  |  |  |  | FDG : muscles (normal CPK) |
| Dastgheyb[128] | 2023 | RP | 29 | F | Bilateral facial palsy (?) then optic tract inflammation | CN | R optic tract lesion |  |  | normal | normal | none | Cs, Cyc | yes |  | brain angiography: normal |
| Gunasekera[129] | 2023 | RP | 53 | M | headache, confusion, left arm weakness | pachyM (no-ANCA) |  |  |  |  |  |  | Cs, TCZ, adalimumab, Cyc | yes | yes |  |
| Husein[130] | 2023 | RP | 70 | F | confusion, urinary retention, TIA | ME | medial temporal, striatal, and WM | nd | yes | 20 | 1.06 |  | CS, Aza | yes |  |  |
| Lee B[131] | 2023 | RP | 58 | F | sub acute side weakness, then hallucinations | ME, myositis | WM and basal ganglia, and spinal cord | LM and perivascular enh. |  | 153 (72) then eosinophilic | 0.61 |  | Cs | no | death | pathology: auricular: chondritis; muscle: vasculitis |
| Michalaki[132] | 2023 | RP | 71 | M | cognitive decline, ataxia | ME | bitemporal, parietal WM lesions | none | yes temporal | 118 (83) | 1 | IgG index, OCB+ | Cs, Cyc, AZA | yes partial | no |  |
| Robert[133] | 2023 | VEXAS | 74 | M | confusion, gait | ME | periventricular WM |  |  | 8 | 0.69 |  | Cs | yes |  |  |
| Valor-Mendez[134] | 2023 | VEXAS | 81 | M | seizure | seizure (isolated) | normal (microangiopathy) |  |  |  |  |  |  |  |  |  |
| Zhang[135] | 2023 | RP | 48 | M | seizure then transient hemiparesis and aphasia | E | none? | diffuse LM |  | 2 | 0.7 |  | Cs | yes | no | COVID infection |
| Abumanhal[136] | 2024 | VEXAS |  |  | Motor neuropathy | PNP |  |  |  |  |  |  |  |  |  |  |
| Abumanhal[136] | 2024 | VEXAS |  |  | Myositis | myositis |  |  |  |  |  |  |  |  |  |  |
| Abumanhal[136] | 2024 | VEXAS |  |  | Confusion | confusion? |  |  |  |  |  |  |  |  |  |  |
| Abumanhal[136] | 2024 | VEXAS |  |  | Cerebral vein thrombosis and seizures | stroke |  |  |  |  |  |  |  |  |  |  |
| Agwan[137] | 2024 | VEXAS | 73 | M | orbital cellulitis | ocular | orbital cellulitis |  |  |  |  |  |  |  |  |  |
| Archambeaud  [138] | 2024 | VEXAS | 68 | M | trismus | myositis | myositis and peripharyngeal cellulitis |  |  |  |  |  | CS | yes | no |  |
| Boret[139] | 2024 | VEXAS | 76 | M | LL myositis, skin lesion | myositis |  |  |  |  |  |  | Cs, MMF, Leflu, Aza, colchicine, | yes | many relapses (SNHL, pulmonary) |  |
| Kaya[140] | 2024 | RP | 49 | F | tetraparesis, coma | stroke | multifocal stroke with haemorrhage: bilateral perforating arteries, pons |  |  | 0 | normal |  | CS, IVIG, infliximab | stabilized | no | Renal cancer. angiography: normal |
| Kikuchi[141] | 2024 | VEXAS ? | 68 | M | headache | pachyM (no-ANCA) |  | left convexity pachyM |  | normal | normal |  | Cs | yes | death (non-neuro) |  |
| Lim[142] | 2024 | VEXAS | 72 | M | calf myositis (normal CK), pseudocellulitis | myositis | limb: myositis |  |  |  |  |  | Cs | nd |  |  |
| Zisapel[143] | 2024 | VEXAS | 82 | M | acute confusion, neglect, seizure | thrombosis | haemorrhage, edema | extended CSVT |  |  |  |  | CS, TCZ | no | death |  |
| Bergonzi[144] | 2025 | VEXAS | 69 | M | comatose during severe bout | E ADEM-like | cerebellar peduncles, corpus callosum, WM, subcortical, round lesions. Halo restricted diffusivity | minor central and peripheral (open-ring) foci |  | none (postCs?) | 1 | none | Cs, Anakinra | no | death | negative antiMOG/AQP4 |
| Bert-Marcaz[145] | 2025 | VEXAS | 60 |  | L LL weakness (monoNP) | PNP |  |  |  | normal | 0.5 |  |  |  |  |  |
| Bert-Marcaz[145] | 2025 | VEXAS | 60 |  | LL Sensory symptoms (axonal PNP) | PNP |  |  |  |  |  |  |  |  |  |  |
| Bert-Marcaz[145] | 2025 | VEXAS | 60 |  | Ataxia, SNHL (VIII), then diplopia, ptosis (III) | CN |  |  |  | normal | 1.2 |  |  |  |  |  |
| Bert-Marcaz[145] | 2025 | VEXAS | 60 |  | LL weakness (axonal SM PNP) | PNP |  |  |  | normal |  |  |  |  |  |  |
| Bert-Marcaz[145] | 2025 | VEXAS | 80 |  | R LL weakness (peroneal axonal monoNP) | PNP | non-specific WM lesions |  |  | normal | 0.5 |  |  |  |  |  |
| Bert-Marcaz[145] | 2025 | VEXAS | 70 |  | diplopia (VI), retro-ocular pain | CN | non-specific WM lesions |  |  |  |  |  |  |  |  |  |
| Bert-Marcaz[145] | 2025 | VEXAS | 70 |  | Four limbs sensitive and motor (demyelin. PNP) | PNP |  |  |  | normal | 0.8 |  |  |  |  |  |
| Bert-Marcaz[145] | 2025 | VEXAS | 60 |  | Confusion, cognitive | E, pachyM | multiple WM lesions | pachyM |  | normal | 0.5 |  |  |  |  |  |
| Bert-Marcaz[145] | 2025 | VEXAS | 80 |  | ataxia | E | multiple WM lesions, then strokes |  |  | normal | 1 |  |  |  |  |  |
| Bert-Marcaz[145] | 2025 | VEXAS | 80 |  | headache, diplopia, then red left eye, intraconal fat infiltration, optic perineuritis | CN | non-specific WM lesions |  |  | normal | 0.5 |  |  |  |  |  |
| Bert-Marcaz[145] | 2025 | VEXAS | 60 |  | LL sensory motor symptoms (axonal non-length PNP) | PNP |  |  |  |  |  |  |  |  |  |  |
| Bert-Marcaz[145] | 2025 | VEXAS | 60 |  | LL+UL sensory motor symptoms (demyelin. Multiple monoNP with blocks) | PNP |  |  |  | normal | 0.7 |  |  |  |  |  |
| Bert-Marcaz[145] | 2025 | VEXAS | 80 |  | LL sensory motor symptoms (axonal PNP) | PNP |  |  |  |  |  |  |  |  |  |  |
| Bert-Marcaz[145] | 2025 | VEXAS | 70 |  | LL sensory ssymptoms (axonal sensory PNP) | PNP |  |  |  |  |  |  |  |  |  |  |
| Bert-Marcaz[145] | 2025 | VEXAS | 70 |  | cognitive impairment, ataxia, then same relapse | E, PRES | periventricular WM, then PRES |  |  | 10 | 1 |  |  |  |  |  |
| Bert-Marcaz[145] | 2025 | VEXAS | 60 |  | LL sensory (axonal non-length PNP) | PNP |  |  |  |  |  |  |  |  |  |  |
| Bert-Marcaz[145] | 2025 | VEXAS | 70 |  | LL+UL sensory (axonal sensory 4 limbs PNP) | PNP |  |  |  |  |  |  |  |  |  |  |
| Bert-Marcaz[145] | 2025 | VEXAS | 60 |  | aphasia, then blindness, then confusion, ataxia | stroke, E, PRES | strokes, and WM lesions |  |  | normal | 0.5 |  |  |  |  |  |
| Bert-Marcaz[145] | 2025 | VEXAS | 70 |  | headache, blindness, then ocular pain, pailledema (optic perineuritis), then L motor weakness | PRES, CN, stroke | periventricular WM lesions, stroke |  |  |  |  |  |  |  |  |  |
| Bert-Marcaz[145] | 2025 | VEXAS | 60 |  | LL+UL sensory (axonal PNP) | PNP |  |  |  |  |  |  |  |  |  |  |
| Bert-Marcaz[145] | 2025 | VEXAS | 70 |  | LL+UL motor and sensory (axonal non-length PNP) | PNP |  |  |  | normal | 0.7 |  |  |  |  |  |
| Bert-Marcaz[145] | 2025 | VEXAS | 80 |  | LL sensory (axonal non-length PNP) | PNP | normal |  |  |  |  |  |  |  |  |  |
| Bert-Marcaz[145] | 2025 | VEXAS | 70 |  | cognitive impairment | E | non-specific WM lesions |  |  |  |  |  |  |  |  |  |
| Bert-Marcaz[145] | 2025 | VEXAS | 70 |  | LL+UL sensory motor symptoms (axonal non-length PNP) | PNP |  |  |  |  |  |  |  |  |  |  |
| Bert-Marcaz[145] | 2025 | VEXAS | 60 |  | ataxia (axonal sensory PNP LL) | PNP |  |  |  |  |  |  |  |  |  |  |
| Bert-Marcaz[145] | 2025 | VEXAS | 70 |  | L motor weakness | stroke | strokes |  |  |  |  |  |  |  |  |  |
| Bert-Marcaz[145] | 2025 | VEXAS | 70 |  | diplopia, ocular pain, headache (VI) | CN |  |  |  |  |  |  |  |  |  |  |
| Bert-Marcaz[145] | 2025 | VEXAS | 70 |  | headache, diplopia (VI), then ataxia (VIII), then facial pain (V) | CN | normal |  |  |  |  |  |  |  |  |  |
| Bert-Marcaz[145] | 2025 | VEXAS | 70 |  | LL sensory (axonal sensory PNP) | PNP |  |  |  |  |  |  |  |  |  |  |
| Bert-Marcaz[145]; Langlois[146] | 2025 | VEXAS | 80 |  | Confusion, cognitive, speech disorder, ataxia | E | multifocal WM and subcortical lesions | minute enhancement | no | 1 | 0.52 | no ITS | Cs, IVIG, ruxolitinib, TCZ | yes (complete MRI remission) | death | (normal TOF, punctate SWI lesions) |
| Caceres-Nazario[147] | 2025 | VEXAS | 71 | M | pain, tremor, altered mental status | E | normal |  |  | normal |  |  | Cs, TCZ | yes | no |  |
| Devaux[148] | 2025 | VEXAS-like | 80 | M | axonal sensori-motor polyneuropathy | PNP |  |  |  |  |  |  | Cs, azacitidine | nd |  |  |
| Han[149] | 2025 | VEXAS | 73 | M | stroke after stopping Cs | stroke | nd |  |  | nd |  |  | none |  | death |  |
| Ocampo-Piraquive[150] | 2025 | RP | 71 | M | 3 attacks aseptic meningitis | M | diffuse WM lesions | diffuse LM enh. Nodular |  | meningitis |  |  | Cs, MTX | yes |  | Meningeal biopsy: lymphocytic meningitis |
| Sullivan[151] | 2025 | VEXAS +PAN |  |  | sensory neuropathy | PNP |  |  |  |  |  |  |  |  |  |  |
| Tagushi[152] | 2025 | RP | 67 | F | confusion | ME | unclear | LM enh. Diffuse |  | 23 (Ly) |  | IgG index, OCB | Cs, MTX | yes | no |  |
| Tshuchida[153] | 2025 | VEXAS | 74 | M | stroke during resuscitation for cardiac arrest during sepsis and bout | stroke |  |  |  |  |  |  |  |  | death |  |
| Tshuchida[153] | 2025 | VEXAS | 69 | M | headache, GCA | GCA |  |  |  |  |  |  | Cs, AZP | improved | no | CT scan: wall thickening of common carotid and subclavian arteries |
| Tshuchida[153]; Kunishita[154] | 2025 | VEXAS | 66 | M | headache, meningitis | M |  |  |  |  |  |  | Cs, TCZ | yes | yes |  |
| Yasuda[155] | 2025 | RP | 48 | M | transient limb sensory motor weakness, headache | pachyM | meninges | diffuse lepto/pachyM |  | 36 (80), 951 (81 PM) | 0.59 to 1.3 | no | IVIG, Cs, infliximab | yes | no | diffusion: restriction fronto-parietal. Episode of bacterial meningitis (P. acnes). Meningeal biopsy: inflammation |
| Ziliotti[156] | 2025 | VEXAS | 52 | M | temporal venous phlebitis | cranial phlebitis |  |  |  |  |  |  |  |  |  |  |

**References**

1. Rucker CW, Ferguson RH. Ocular manifestations of relapsing polychondritis. *Arch Ophthalmol*. 1965; 73:46–48.

2. Herman JH, Dennis MV. Immunopathologic Studies in Relapsing Polychondritis. *J Clin Invest*. 1973; 52(3):549–558.

3. McAdam LP, O’Hanlan MA, Bluestone R, Pearson CM. Relapsing polychondritis: prospective study of 23 patients and a review of the literature. *Medicine (Baltimore)*. 1976; 55(3):193–215.

4. Ridgway HB, Hansotia PL, Schorr WF. Relapsing polychondritis: unusual neurological findings and therapeutic efficacy of dapsone. *Arch Dermatol*. 1979; 115(1):43–45.

5. Sundaram MBM, Rajput AH. Nervous system complications of relapsing polychondritis. *Neurology*. 1983; 33(4):513–513.

6. Hull RG, Morgan SH. The nervous system and relapsing polychondritis. *Neurology*. 1984; 34(4):557.

7. Willis J, Atack EA, Kraag G. Relapsing polychondritis with multifocal neurological abnormalities. *Can J Neurol Sci*. 1984; 11(3):402–404.

8. Serratrice G, Pouget J, Saint-Jean JC. [Sensory trigeminal neuropathy in systemic diseases: 4 cases with study of the trigeminofacial reflex]. *Rev Neurol (Paris)*. 1986; 142(5):535–540.

9. Brod S, Booss J. Idiopathic CSF pleocytosis in relapsing polychondritis. *Neurology*. 1988; 38(2):322–323.

10. Morita T, Hashimoto T, Uozumi T, *et al.* Relapsing polychondritis with neurological signs similair to dentatorubropallidoluysian atrophy. *J. Jpn. Soc. Intern. Med.* 1988; 77(4):516–519.

11. Stewart SS, Ashizawa T, Dudley AW, Goldberg JW, Lidsky MD. Cerebral vasculitis in relapsing polychondritis. *Neurology*. 1988; 38(1):150–152.

12. Schindzielorz A, Edberg SC, Bia FJ. Strongyloides stercoralis Hyperinfection and Central Nervous System Involvement in a Patient With Relapsing Polychondritis: *Southern Medical Journal*. 1991; 84(8):1055–1057.

13. Strobel ES, Lang B, Schumacher M, Peter HH. Cerebral aneurysm in relapsing polychondritis. *J Rheumatol*. 1992; 19(9):1482–1483.

14. Wasserfallen JB, Schaller MD. Unusual rhombencephalitis in relapsing polychondritis. *Annals of the Rheumatic Diseases*. 1992; 51(10):1184.

15. Bouton R, Capon A. Stroke as initial manifestation of relapsing polychondritis. *Ital J Neurol Sci*. 1994; 15(1):61–63.

16. Hanslik T, Wechsler B, Piette JC, *et al.* Central nervous system involvement in relapsing polychondritis. *Clin Exp Rheumatol*. 1994; 12(5):539–541.

17. Berg AM, Kasznica J, Hopkins P, Simms RW. Relapsing polychondritis and aseptic meningitis. *J Rheumatol*. 1996; 23(3):567–569.

18. Ragnaud JM, Tahbaz A, Morlat P, *et al.* Recurrent Aseptic Purulent Meningitis in a Patient with Relapsing Polychondritis. *Clinical Infectious Diseases*. 1996; 22(2):374–374.

19. Kothare SV, Chu C-C, VanLandingham K, *et al.* Migratory leptomeningeal inflammation with relapsing polychondritis. *Neurology*. 1998; 51(2):614–617.

20. Ramos JM, Blázquez RM, Climent A, Peña MA. [Aseptic meningitis, erythema nodosum and centrifugal annular erythema as first manifestation of recurrent polychondritis]. *Med Clin (Barc)*. 2000; 114(5):196–197.

21. Dreher A, Aigner J, Fuchshuber S, Kastenbauer E. Relapsing Polychondritis: A Course Over 20 Years With Cerebral Involvement. *Arch Otolaryngol Head Neck Surg*. 2000; 126(12):1495.

22. Coumbaras M, Boulin A, Piette AM, *et al.* Intracranial aneurysm associated with relapsing polychondritis. *Neuroradiology*. 2001; 43(7):565–566.

23. Brucki SMD, Rocha MSG. Policondrite recidivante associada a meningoencephalite. *Arq. Neuro-Psiquiatr.* 2001; 59(3A).

24. Sost G, Noyon V, Cador B, Jégo P, Grosbois B. Polychondrite atrophiante et méningoencéphalite aseptique. *La Revue de Médecine Interne*. 2001; 22(7):673–675.

25. Yamazaki K, Suga T, Hirata K. Large vessel arteritis in relapsing polychondritis. *J Laryngol Otol*. 2001; 115(10):836–838.

26. Elmaci I, Altinoz MA, Akdemir G, *et al.* Neurosurgical and neuro-immunological management of IgG4-related hypertrophic sclerosing pachymeningitis. A literature survey and discussion of a unique index case. *Clin Neurol Neurosurg*. 2021; 200:106342.

27. Hosford I, Glass J, Baker N. Relapsing polychondritis--an unusual but potentially treatable cause of cognitive impairment. *N Z Med J*. 2003; 116(1175):U463.

28. Topalkara K, Kaptanoglu E, Akyüz A, Yurtçu S, Akman V. Relapsing polychondritis with involvement of posterior inferior cerebellar artery causing acute lateral medullary syndrome. *J Clin Rheumatol*. 2003; 9(2):92–95.

29. Fujiki F. Non-herpetic limbic encephalitis associated with relapsing polychondritis. *Journal of Neurology, Neurosurgery & Psychiatry*. 2004; 75(11):1646–1647.

30. Gertner E. Severe recurrent neurological disease in the MAGIC syndrome. *J Rheumatol*. 2004; 31(5):1018–1019.

31. Ohta Y, Nagano I, Niiya D, *et al.* Nonparaneoplastic limbic encephalitis with relapsing polychondritis. *Journal of the Neurological Sciences*. 2004; 220(1–2):85–88.

32. Yang S-M, Chou C-T. Relapsing Polychondritis With Encephalitis: *JCR: Journal of Clinical Rheumatology*. 2004; 10(2):83–85.

33. Ota M, Mizukami K, Hayashi T, Sumida T, Asada T. Brain magnetic resonance imaging and single photon emission computerized tomography findings in a case of relapsing polychondritis showing cognitive impairment and personality changes. *Progress in Neuro-Psychopharmacology and Biological Psychiatry*. 2005; 29(2):347–349.

34. Head E, Starr A, Kim RC, *et al.* Relapsing polychondritis with features of dementia with Lewy bodies. *Acta Neuropathol*. 2006; 112(2):217–225.

35. Hsu K-C, Wu Y-R, Lyu R-K, Tang L-M. Aseptic meningitis and ischemic stroke in relapsing polychondritis. *Clin Rheumatol*. 2006; 25(2):265–267.

36. Irani SR, Soni A, Beynon H, Athwal BS. Relapsing encephalo polychondritis. *Pract. neurol.* 2006; 6(6):372–375.

37. Sato K, Kubota T, Kitai R, Miyamori I. Meningeal plasma cell granuloma with relapsing polychondritis. *JNS*. 2006; 104(1):143–146.

38. Yan M, Harper C, Schwartz R. Dementia in a patient with non-paraneoplastic limbic encephalitis associated with relapsing polychondritis. *Pathology*. 2006; 38(6):596–599.

39. Choi J-Y. A Case of Meningoencephalitis Associated with Relapsing Polychondritis. *Journal of the Korean Neurological Association*. 2007; 25(2):213–217.

40. Hirunwiwatkul P, Trobe JD. Optic neuropathy associated with periostitis in relapsing polychondritis. *J Neuroophthalmol*. 2007; 27(1):16–21.

41. Baaj AA, Vale FL, Carter JD, Rojiani AM. Granulomatosis with CNS involvement: a neuroimaging clinicopathologic correlation. *J Neuroimaging*. 2009; 19(2):194–197.

42. Erten-Lyons D, Oken B, Woltjer RL, Quinn J. Relapsing polychondritis: an uncommon cause of dementia. *Journal of Neurology, Neurosurgery & Psychiatry*. 2008; 79(5):609–610.

43. Fujioka S, Tsuboi Y, Mikasa M, *et al.* A case of encephalitis lethargica associated with relapsing polychondritis. *Movement Disorders*. 2008; 23(16):2421–2423.

44. Kuwabara M, Shimono T, Toyomasu M, *et al.* “Prominent ear sign” on diffusion-weighted magnetic resonance imaging in relapsing polychondritis. *Radiat Med*. 2008; 26(7):438–441.

45. Andrade-Ramos M, Avila-Carrillo A, Navarro-Bonnet J, *et al.* Encefalo-policondritis recidivante. Reporte de dos casos. *Revista Mexicana de Neurociencia*. 2009; 10(4):304–8.

46. Imamura E, Yamashita H, Fukuhara T, *et al.* [Autopsy case of perivasculitic meningoencephalitis associated with relapsing polychondritis presenting with central nervous system manifestation]. *Rinsho shinkeigaku = Clinical neurology*. 2009; 49(4). Available at: https://pubmed.ncbi.nlm.nih.gov/19462815/ [Accessed June 2, 2025].

47. Kashihara K, Kawada S, Takahashi Y. Autoantibodies to glutamate receptor GluRε2 in a patient with limbic encephalitis associated with relapsing polychondritis. *Journal of the Neurological Sciences*. 2009; 287(1–2):275–277.

48. Kumar N, Leep Hunderfund AN, Kutzbach BR, Pulido JS, Miller GM. A Limbic Encephalitis MR Imaging in a Patient with Behçet Disease and Relapsing Polychondritis: Fig 1. *AJNR Am J Neuroradiol*. 2009; 30(7):e96–e96.

49. Pamuk ON, Harmandar F, Cakir N. The development of trigeminal neuralgia related to auricular chondritis in a patient with rheumatoid arthritis-relapsing polychondritis and its treatment with etanercept. Description of the first case. *Clin Exp Rheumatol*. 2009; 27(1):128–129.

50. Swen SJ, Leonards DJH, Swen W a. A, de Jonghe JFM, Kalisvaarte KJ. [Reversible cognitive decline in a patient with relapsing polychondritis]. *Tijdschr Gerontol Geriatr*. 2009; 40(5):203–207.

51. Yaguchi H, Tsuzaka K, Niino M, Yabe I, Sasaki H. Aseptic Meningitis with Relapsing Polychondritis Mimicking Bacterial Meningitis. *Intern. Med.* 2009; 48(20):1841–1844.

52. Sampaio L, Silva L, Mariz E, Ventura F. Central nervous system involvement in relapsing polychondritis. *Joint Bone Spine*. 2010; 77(6):619–620.

53. Cañas CA, Díaz-Martínez JC, Tobón GJ. Combination of hypertrophic pachymeningitis, PR3-ANCA-positive vasculitis, and relapsing polychondritis. *J Rheumatol*. 2011; 38(5):966–967.

54. Choi HJ, Lee HJ. Relapsing Polychondritis With Encephalitis. *JCR: Journal of Clinical Rheumatology*. 2011; 17(6):329–331.

55. Garcia-Egido A, Gutierrez C, De la Fuente C, Gomez F. Relapsing polychondritis-associated meningitis and encephalitis: response to infliximab. *Rheumatology (Oxford)*. 2011; 50(9):1721–1723.

56. Roux C, Guey S, Crassard I, *et al.* A rare cause of gait ataxia. *Lancet*. 2011; 378(9798):1274.

57. Storey K, Matěj R, Rusina R. Unusual association of seronegative, nonparaneoplastic limbic encephalitis and relapsing polychondritis in a patient with history of thymectomy for myasthenia: a case study. *J Neurol*. 2011; 258(1):159–161.

58. Wang ZJ, Pu CQ, Wang ZJ, *et al.* Meningoencephalitis or meningitis in relapsing polychondritis: Four case reports and a literature review. *Journal of Clinical Neuroscience*. 2011; 18(12):1608–1615.

59. Defer G, Danaila T, Constans JM, Derache N. Relapsing polychondritis revealed by basal ganglia lesions. *Movement Disorders*. 2012; 27(9):1094–1096.

60. Prinz S, Dafotakis M, Schneider R, Mühlenbruch G, Stopschinski B. Das „red puffy ear sign“ - ein klinisches Zeichen zur Diagnose einer seltenen Meningoenzephalitis. *Fortschr Neurol Psychiatr*. 2012; 80(08):463–467.

61. Ducci RD-P, Germiniani FMB, Czecko LEA, Paiva ES, Teive HAG. Relapsing polychondritis and lymphocytic meningitis with varied neurological symptoms. *Revista Brasileira de Reumatologia (English Edition)*. 2017; 57(6):623–625.

62. Çoban EK, Xanmemmedov E, Çolak M, Soysal A. A RARE COMPLICATION OF A RARE DISEASE; STROKE DUE TO RELAPSING POLYCHONDRITIS. *Ideggyogy Sz*. 2015; 68(11–12):429–432.

63. Mattiassich G, Egger M, Semlitsch G, Rainer F. Occurrence of relapsing polychondritis with a rising cANCA titre in a cANCA-positive systemic and cerebral vasculitis patient. *BMJ Case Rep*. 2013; 2013:bcr2013008717, bcr-2013–008717.

64. Moemoe AC, Kim TE, Kim HJ, Park SA. A Case of Meningoencephalitis due to Relapsing Polychondritis Presenting Cognitive Impairment; Serial Neuropsychological Tests. *Dementia and Neurocognitive Disorders*. 2013; 12(2):56–59.

65. Hatti K, Giuliano V. Central Nervous System Involvement in Relapsing Polychondritis: *JCR: Journal of Clinical Rheumatology*. 2014; 20(7):396–397.

66. Kondo T, Fukuta M, Takemoto A, *et al.* Limbic encephalitis associated with relapsing polychondritis responded to infliximab and maintained its condition without recurrence after discontinuation: a case report and review of the literature. *Nagoya J. Med. Sci*. 2014; 76:361)368.

67. Nishiguchi R, Fujimoto T, Eguchi K, Fukuda Y, Takahashi Y. [A case of bilateral auricular chondritis with anti-glutamate receptor (GluRε2) antibody-positive non-herpetic acute limbic encephalitis]. *Rinsho Shinkeigaku*. 2015; 55(6):395–400.

68. Niwa A, Okamoto Y, Kondo T, *et al.* Perivasculitic Panencephalitis with Relapsing Polychondritis: An Autopsy Case Report and Review of Previous Cases. *Intern. Med.* 2014; 53(11):1191–1195.

69. Baba T, Kanno S, Shijo T, *et al.* Callosal Disconnection Syndrome Associated with Relapsing Polychondritis. *Intern. Med.* 2016; 55(9):1191–1193.

70. Jeon CH. Relapsing Polychondritis with Central Nervous System Involvement: Experience of Three Different Cases in a Single Center. *J Korean Med Sci*. 2016; 31(11):1846.

71. Hwang Y-P, Kuo R, Chen T-L, Chen P-H, Cheng S-J. Relapsing Polychondritis Presenting with Meningoencephalitis and Dementia: Correlation with Neuroimaging and Clinical Features. 2015; 24(1).

72. Karapanayiotides T, Kouskouras K, Ioannidis P, *et al.* Internal Carotid Artery Floating Thrombus in Relapsing Polychondritis. *Journal of Neuroimaging*. 2015; 25(1):142–144.

73. Nakamura K, Sugaya K, Nakata Y, *et al.* Hypertrophic pachymeningitis and encephalitis in a patient with relapsing polychondritis. *Neurology and Clinical Neuroscience*. 2015; 3(1):42–43.

74. Nara M, Komatsuda A, Togashi M, Wakui H. Relapsing polychondritis with encephalitis: a case report and literature review. *Intern Med*. 2015; 54(2):231–234.

75. Almackenzie M, Alharbi A, Alhassan S, Cook E, Altorok N. Successful Treatment of Central Nervous System Vasculitis Associated with Relapsing Polychondritis With Cyclophosphamide. *Am J Med Sci*. 2017; 353(5):495–497.

76. Chernyak VI, Savel’ev A I null, Men’shikova IV, Pogromov AP. Nervous system lesions associated with relapsing polychondritis : analysis of original observations. *Klin Med (Mosk)*. 2016; 94(2):108–113.

77. Dion J, Costedoat‐Chalumeau N, Sène D, *et al.* Relapsing Polychondritis Can Be Characterized by Three Different Clinical Phenotypes: Analysis of a Recent Series of 142 Patients. *Arthritis & Rheumatology*. 2016; 68(12):2992–3001.

78. Ellis RJB, Mbizvo GK, Jacob A, Doran M, Larner AJ. Relapsing polychondritis complicated by cognitive dysfunction: two distinct clinical phenotypes? *International Journal of Neuroscience*. 2017; 127(2):124–134.

79. Eun YH, Seok H, Shin IS, *et al.* Relapsing polychondritis presenting with inflammatory pseudotumor. *Korean J Intern Med*. 2016; 31(5):1003–1005.

80. Kamboj AK, Cotter TG, Varghese C. Relapsing Polychondritis with Myelodysplastic Syndrome: A Case Report. *The American Journal of Medicine*. 2017; 130(3):e107–e108.

81. Kawabe M, Tachi R, Hideyama T, *et al.* Relapsing polychondritis-associated limbic encephalitis preceding the emergence of systemic inflammation. *Neurology and Clinical Neuroscience*. 2016; 4(4):162–164.

82. Lin D-F, Yang W-Q, Zhang P-P, *et al.* Clinical and prognostic characteristics of 158 cases of relapsing polychondritis in China and review of the literature. *Rheumatol Int*. 2016; 36(7):1003–1009.

83. Liu L, Liu S, Guan W, Zhang L. Efficacy of tocilizumab for psychiatric symptoms associated with relapsing polychondritis: the first case report and review of the literature. *Rheumatol Int*. 2016; 36(8):1185–1189.

84. Maillet T, Vinit J, Rouland A, Gandon C, Mausservey C. [Relapsing polychondritis with meningitis: Focus on a case report demonstrating remission under immunosuppressive agents]. *Presse Med*. 2016; 45(10):938–939.

85. Simabukuro MM, Lucato LT, Shinjo SK, Flores WL, Castro LHM. Teaching Neuro *Images* : Limbic encephalitis associated with relapsing polychondritis. *Neurology*. 2016; 86(20). Available at: https://www.neurology.org/doi/10.1212/WNL.0000000000002677 [Accessed May 22, 2025].

86. Ushiyama S, Kinoshita T, Shimojima Y, *et al.* Hypertrophic Pachymeningitis as an Early Manifestation of Relapsing Polychondritis: Case Report and Review of the Literature. *Case Rep Neurol*. 2016; 8(3):211–217.

87. Ahn S-W, Park M-S, Jeong H-B, *et al.* Relapsing Polychondritis Presented with Encephalitis Followed by Brain Atrophy. *Exp Neurobiol*. 2017; 26(1):66–69.

88. Ismail FS, Enzi B, Boy C, *et al.* Relapsing polychondritis as an unusual cause of multiple cranial nerve palsies—A Case report. *Muscle and Nerve*. 2017; 56(1). Available at: https://onlinelibrary.wiley.com/doi/10.1002/mus.25582 [Accessed May 22, 2025].

89. Le Marec J, Jobard S, Bigot A, *et al.* Letter to the Editor: Central Nervous System Involvement in Relapsing Polychondritis, a Rare and Difficult Diagnosis: a Case Report. *J Korean Med Sci*. 2017; 32(6):1048.

90. Ma B, Wu H, Yin H, *et al.* Management of hydrocephalus associated with autoimmune diseases: a series of 19 cases. *Autoimmunity*. 2017; 50(7):422–427.

91. Shen K, Yin G, Yang C, Xie Q. Aseptic meningitis in relapsing polychondritis: a case report and literature review. *Clin Rheumatol*. 2018; 37(1):251–255.

92. Tsai M, Hu M, Zussman J, Worswick S. Relapsing polychondritis with meningoencephalitis. *Cutis*. 2017; 99(1):43–46.

93. Zhou A. Relapsing Polychondritis with Central Nervous System Involvement Presented as Encephalitis: 7 Cases and Literature Review. *Journal of Rheumatic Diseases and Treatment*. 2017; 3(1). Available at: https://clinmedjournals.org/articles/jrdt/journal-of-rheumatic-diseases-and-treatment-jrdt-3-045.php?jid=jrdt [Accessed June 2, 2025].

94. Cao J, Zhang M. Pleocytosis in a patient with relapsing polychondritis accompanied by meningoencephalitis: a case report. *BMC Neurology*. 2018; 18(1):53.

95. Al-Tabbaa MM, Habal H. Relapsing Polychondritis with Meningoencephalitis Refractory to Immunosuppressant Therapy. *Case Rep Neurol Med*. 2018; 2018:1873582.

96. Shashikala TP, Mehta S, Sharma A, Rai Mittal B. Multiple Cranial-Nerve Palsies: An Unusual Culprit. *J Clin Neurol*. 2018; 14(2):257–258.

97. Zhu Z, Tian D, Ren N, *et al.* Limbic encephalitis with relapsing polychondritis: persistent white matter lesions and brain atrophy. *J Int Med Res*. 2018; 46(12):5297–5302.

98. Miyano R, Kurihara M, Orimo K, *et al.* Severe visual impairment and subclinical encephalitis preceding clinical signs of chondritis in relapsing polychondritis. *Neurology and Clinical Neuroscience*. 2019; 7(2):75–77.

99. Anada R, Hayashi T, Yamamoto M, Nakatsuji Y. The Spinal Cord Uptake of Fluorodeoxyglucose in a Patient with Relapsing Polychondritis. *Intern. Med.* 2020; 59(10):1339–1340.

100. Angkodjojo S, Yeo CJJ. A Patient with Limbic Encephalitis, Ear Perichondritis, and Episcleritis – An Unusual Presentation of Relapsing Polychondritis. *Case Rep Neurol*. 2020; 12(3):378–386.

101. Bravo-Ruiz OL, García-Ortega YE, Salcedo-Hernández MDJ, Mireles-Ramírez MA, González-Usigli HA. Encefalitis límbica secundaria a policondritis recidivante. *El Residente*. 2020; 15(3):113–117.

102. Chaucer B, Demanes A, Stone A, Kakollu V. Hemorrhagic Stroke in Relapsing Polychondritis: A Rare Complication of a Rare Disease. *Case Reports in Rheumatology*. 2020; 2020:1–3.

103. Farhat R, Clavel G, Villeneuve D, *et al.* Sustained Remission with Tocilizumab in Refractory Relapsing Polychondritis with Ocular Involvement: A Case Series. *Ocul Immunol Inflamm*. 2021; 29(1):9–13.

104. Ikeda T, Asano M, Kitada Y, *et al.* Relapsing Polychondritis with a Cobble-stone Appearance of the Tracheal Mucosa, Preceded by Posterior Reversible Encephalopathy Syndrome. *Intern. Med.* 2020; 59(8):1093–1097.

105. Lin H, Huang Y, Zeng H, *et al.* Overlapping Clinical Syndromes in Patients with Glial Fibrillary Acidic Protein IgG. *Neuroimmunomodulation*. 2020; 27(1):69–74.

106. Matsuzono K, Furuya K, Igarashi T, *et al.* Relapsing polychondritis coupling with cerebral amyloid deposit inducing cerebral amyloid angiopathy-related inflammation. *J Thromb Thrombolysis*. 2020; 49(4):681–684.

107. Cao X, Zhu L, Li H, *et al.* Comparison of relapsing polychondritis patients with and without central nervous system involvement: A retrospective study of 181 patients. *Int J Immunopathol Pharmacol*. 2021; 35:20587384211000547.

108. Contreras D, Dhillon N, Sharma R, *et al.* When You “Can’t See” a Case of Relapsing Polychondritis. *Journal of Investigative Medicine High Impact Case Reports*. 2021; 9:23247096211052175.

109. Cordts I, Hecker JS, Gauck D, *et al.* Successful treatment with azacitidine in VEXAS syndrome with prominent myofasciitis. *Rheumatology*. 2022; 61(5):e117–e119.

110. Fan T, Chima J, George P, Newey C. Stroke and Vasculitis, a rare but serious complication of Relapsing Polychondritis: A Case Report and Literature Review (4900). *Neurology*. 2021; 96.

111. Hutto SK, Maher MD, Miloslavsky EM, Venna N. Nodular Pachymeningitis Associated With Relapsing Polychondritis and Crohn Disease Responsive to Adalimumab and Prednisone. *Neurol Neuroimmunol Neuroinflamm*. 2021; 8(5):e1022.

112. Matsumoto H, Tokimura R, Fujita Y, *et al.* Meningoencephalitis in relapsing polychondritis: A case report. *Medicine (Baltimore)*. 2021; 100(24):e26315.

113. Obiorah IE, Patel BA, Groarke EM, *et al.* Benign and malignant hematologic manifestations in patients with VEXAS syndrome due to somatic mutations in *UBA1*. *Blood Advances*. 2021; 5(16):3203–3215.

114. van der Made CI, Potjewijd J, Hoogstins A, *et al.* Adult-onset autoinflammation caused by somatic mutations in UBA1: A Dutch case series of patients with VEXAS. *J Allergy Clin Immunol*. 2022; 149(1):432-439.e4.

115. Vera C, Matamala JM, Feuerhake W, *et al.* Encefalopatía subaguda en una paciente con policondritis recidivante. Caso clínico. *Rev Med Chile*. 2021; 149:1085–1089.

116. Xu Y, Meng Y, Wang P, Sun L, Xu S. Teaching NeuroImage: Seizures as the Initial Symptom of Relapsing Polychondritis. *Neurology*. 2022; 98(6):e677–e678.

117. Bert-Marcaz C, Briantais A, Faucher B, *et al.* Expanding the spectrum of VEXAS syndrome: association with acute-onset CIDP. *J Neurol Neurosurg Psychiatry*. 2022; 93(7):797–798.

118. Collantes-Rodríguez C, Jiménez-Gallo D, de la Varga-Martínez R, *et al.* Vexas syndrome successfully treated with canakinumab. *J Dtsch Dermatol Ges*. 2023; 21(1):69–70.

119. Escoda T, Farnault L, Gallard J, *et al.* Azacitidine, a therapeutic option in Lewis and Sumner syndrome associated with VEXAS syndrome. *Rev Neurol (Paris)*. 2022; 178(10):1109–1111.

120. Guerrero-Bermúdez CA, Cardona-Cardona AF, Ariza-Parra EJ, *et al.* Vacuoles, E1 enzyme, X-linked, autoinflammatory, somatic syndrome (VEXAS syndrome) with prominent supraglottic larynx involvement: a case-based review. *Clin Rheumatol*. 2022; 41(11):3565–3572.

121. Holmes A, Thant A, Correy R, Vilain R. Inflammatory pseudotumour arising secondary to VEXAS syndrome. *Pathology*. 2023; 55(1):161–163.

122. Lee D, Youn J, Cho JW, Ahn JH. Relapsing polychondritis with multiple neurologic manifestations. *Acta Neurol Belg*. 2022; 122(5):1397–1398.

123. Rachdi M, Beun AJ, Kampouridis S, Willermain F, Buelens T. Focal Pachymeningitis Induced Papilloedema as an Early Manifestation of Relapsing Polychondritis. *Neuroophthalmology*. 2023; 47(3):145–152.

124. Topilow JS, Ospina Cardona D, Beck DB, *et al.* Novel genetic mutation in myositis-variant of VEXAS syndrome. *Rheumatology*. 2022; 61(12):e371–e373.

125. Yokota K, Tachibana H, Miyake A, Yamamoto T, Mimura T. Relapsing Polychondritis and Aseptic Meningoencephalitis. *Intern. Med.* 2023; 62(3):481–486.

126. Yoon JG, Lee S, Kim S, *et al.* The First Korean Case of VEXAS Syndrome Caused by a *UBA1* Somatic Variant. *Ann Lab Med*. 2023; 43(2):217–220.

127. Belicard F, Belhomme N, Bouzy S, *et al.* Vacuoles, E1 enzyme, X-linked, autoinflammatory, and somatic syndrome in the intensive care unit: a case report. *J Med Case Rep*. 2023; 17:314.

128. Dastgheyb N, Kline LB, Dhital R, *et al.* Optic Tract Involvement in a Patient With Relapsing Polychondritis. *J Neuroophthalmol*. 2024; 44(3):e402–e404.

129. Gunasekera L, Ginevra M, Sharma R, Malhotra A. 2689 Hypertrophic pachymeningitis in setting of relapsing polychondronditis – a management issue. *BMJ Neurol Open*. 2024; 5(Suppl 1). Available at: https://neurologyopen.bmj.com/content/5/Suppl_1/A34.2 [Accessed June 15, 2025].

130. Husein S, Murayama Y, Koo A, Wakefield M, Buccoliero R. Relapsing polychondritis presenting with sero-negative limbic encephalitis. *Clinical Medicine*. 2023; 23(6):618–620.

131. Lee B, Peterson C, Peterson J, Lacy M. Relapsing polychondritis presenting as otitis externa. *The Southwest Respiratory and Critical Care Chronicles*. 2023; 11(46):44–52.

132. Michalaki V, Katsifis-Nezis D, Rallis T, Kanavouras K, Tsouris Z. Limbic Encephalitis as a Late Complication of Relapsing Polychondritis: A Case Report and Review of the Literature. *MJR*. 2023; 34(2):229.

133. Robert M, Berleur M, Gaudemer A, *et al.* VEXAS syndrome: Expanding the clinical and molecular spectrum. *Joint Bone Spine*. 2023; 90(3):105531.

134. Valor-Méndez L, Sticherling M, Zeschick M, *et al.* VEXAS syndrome mimicking lupus-like disease. *Rheumatology*. 2023; 62(9):e271–e272.

135. Zhang D, Shi J, Zhang X, Wang J, Shao Y. Relapsing polychondritis-associated meningoencephalitis initially presenting as seizure: a case report and literature review. *Front. Neurol.* 2023; 14:1265345.

136. Abumanhal M, Leibovitch I, Zisapel M, *et al.* Ocular and orbital manifestations in VEXAS syndrome. *Eye (Lond)*. 2024; 38(9):1748–1754.

137. Agwan S, Zhang L-Y, Baker T, *et al.* A vexing case of a 73-year-old man with fevers, orbital cellulitis, and asymptomatic interstitial lung disease. *Respirol Case Rep*. 2024; 12(9):e70020.

138. Archambeaud A, Le Dreau C, Bigot A, *et al.* Trismus as a new feature of VEXAS syndrome. *Rheumatology (Oxford)*. 2024; 63(9):e258–e260.

139. Boret M, Malfait T. Case report: diagnosis of VEXAS syndrome in a patient with therapy-resistant large vessel vasculitis. *Acta Clin Belg*. 2024; 79(2):143–147.

140. Kaya H, Sarıkaya E, Kayım Yıldız Ö, *et al.* Relapsing polychondritis and stroke. *Turkish Journal of Cerebrovascular Diseases*. 2024; 30(1):61–66.

141. Kikuchi S, Hayashi T, Nitta H, *et al.* Cranial hypertrophic pachymeningitis with myelodysplastic syndrome. *Heliyon*. 2024; 10(12):e32973.

142. Lim JWW, Chua CKT. Case report: Lower limb pseudocellulitis due to calf myositis in VEXAS syndrome. *Int J of Rheum Dis*. 2024; 27(7):e15270.

143. Zisapel M, Seyman E, Molad J, *et al.* Case report: Cerebral sinus vein thrombosis in VEXAS syndrome. *Front. Med.* 2024; 11:1377768.

144. Bergonzi GM, Campochiaro C, Tomelleri A, *et al.* VEXAS associated acute disseminated Encephalo-Myelitis (ADEM)-like syndrome: A case report and review of the literature. *Current Research in Translational Medicine*. 2025; 73(3):103505.

145. Bert-Marcaz C, Fortanier É, Briantais A, *et al.* Neurological manifestations in patients with VEXAS syndrome. *J Neurol*. 2025; 272(2):181.

146. Langlois V, Curie A, Demas A, *et al.* Central nervous system vasculitis in VEXAS syndrome: A rare involvemen. *Clinical Neurology and Neurosurgery*. 2024; 242:108351.

147. Cáceres-Nazario B, Rivenbark J, Saha MK, Mathews S, Rubinstein SM. Novel use of Siltuximab in a patient with VEXAS Syndrome. *Ann Hematol*. 2025; 104(2):1259–1267.

148. Devaux M, Jachiet V, Hirsch P, *et al.* Syndromes auto-inflammatoires VEXAS-like : à propos de 2 cas. *La Revue de Médecine Interne*. 2025; 46(3):139–145.

149. Han Z, Fan H, Guo Q, *et al.* Death with cerebral infarction in a patient with VEXAS syndrome. *Rheumatology (Oxford)*. 2025; 64(3):1545–1547.

150. Ocampo-Piraquive V, Aguirre-Valencia D, Delgado-Mora T, Hormaza-Jaramillo A. Recurrent lymphocytic meningitis and progressive dementia: manifestations of relapsing polychondritis: a case report. *BMC Neurol*. 2025; 25(1):171.

151. Sullivan MM, Mead-Harvey C, Sartori-Valinotti JC, *et al.* Vasculitis associated with VEXAS syndrome. *Rheumatology (Oxford)*. 2024; 64(6):3889–3894.

152. Taguchi T, Matsubara S, Nakahara K, Ohmori H, Ueda M. Ear and Nose Abnormalities in Meningoencephalitis Associated With Relapsing Polychondritis: A Case Report. *Cureus*. 2025. Available at: https://www.cureus.com/articles/341668-ear-and-nose-abnormalities-in-meningoencephalitis-associated-with-relapsing-polychondritis-a-case-report [Accessed July 8, 2025].

153. Tsuchida N, Kunishita Y, Uchiyama Y, *et al.* Pathogenic UBA1 variants associated with VEXAS syndrome in Japanese patients with relapsing polychondritis. *Ann Rheum Dis*. 2021; 80(8):1057–1061.

154. Kunishita Y, Kirino Y, Tsuchida N, *et al.* Case Report: Tocilizumab Treatment for VEXAS Syndrome With Relapsing Polychondritis: A Single-Center, 1-Year Longitudinal Observational Study In Japan. *Front. Immunol.* 2022; 13:901063.

155. Yasuda K, Maki T, Nishimura K, *et al.* Unusual Clinical Course in a Case with Relapsing Polychondritis Showing Hypertrophic Pachymeningitis Complicated by a *Klebsiella pneumoniae* Infection. *Intern. Med.* 2025:5334–25.

156. Ziliotti R, Seitz L, Gasser M, Seitz P. Temporal phlebitis in VEXAS syndrome. *Rheumatology*. 2025; 64(1):372–373.
